# Spatially Confined Face‐Selective Growth of Large‐Area 2D Organic Molecular Crystals in a Supramolecular Gel for Highly Efficient Flexible Photodetection

**DOI:** 10.1002/advs.202203662

**Published:** 2022-09-01

**Authors:** Chaowen Shen, Pan Han, Zhi Zheng, Wenhe Jiang, Sheng Gao, Chunxia Hua, Cheng Lung Chen, Fan Xia, Tianyou Zhai, Kaiqiang Liu, Yu Fang

**Affiliations:** ^1^ Key Laboratory of Applied Surface and Colloid Chemistry Ministry of Education School of Chemistry and Chemical Engineering Shaanxi Normal University Xi′an 710119 P. R. China; ^2^ State Key Laboratory of Materials Processing and Die and Mould Technology School of Materials Science and Engineering Huazhong University of Science and Technology Wuhan 430074 P. R. China; ^3^ Engineering Research Center of Nano‐Geomaterials of Ministry of Education Faculty of Materials Science and Chemistry China University of Geosciences Wuhan 430074 P. R. China; ^4^ Department of Chemistry National Sun Yat‐sen University Kaosiung Taiwan 80424 P. R. China

**Keywords:** 2D organic molecular crystals, anti‐solvent vapor diffusion, flexible optoelectronics, photodetectors, supramolecular gel crystallization

## Abstract

2D organic molecular crystals (2DOMCs) are promising materials for the fabrication of high‐performance optoelectronic devices. However, the growth of organic molecules into 2DOMCs remains a challenge because of the difficulties in controlling their self‐assembly with a preferential orientation in solution‐process crystallization. Herein, fullerene is chosen as a model molecule to develop a supramolecular gel crystallization approach to grow large‐area 2DOMCs by controlling the perfect arrangement on the {220} crystal plane with the assistance of a gelated solvent. In this case, the gel networks provide tuneable confined spaces to control the crystallization kinetics toward the growth of dominant crystal faces by their inhibiting motions of solvent or solute molecules to enable the growth of perfect crystals at appropriate nucleation rates. As a result, a large‐area fullerene 2DOMC is produced successfully and its corresponding device on a flexible substrate exhibits excellent bendable properties and ultra‐high weak light detection ability (2.9 × 10^11^ Jones) at a 10 V bias upon irradiation with 450 nm incident light. Moreover, its photoelectric properties remain unchanged after 200 cycles of bending at angles of 45, 90, and 180°. These results can be extended to the growth of other 2DOMCs for potentially fabricating advanced organic (opto)electronics.

## Graphical abstract

3D networks of supramolecular gels provide tuneable spatially confined spaces to drive the growth of large‐area 2D fullerene molecular crystals under controlled supersaturation. This strategy is expected to be an efficient and universal method for the growth of organic semiconductors into 2D organic molecular crystals for their potential applications.

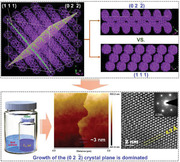

## 1 Introduction

2D organic molecular crystals (2DOMCs) are a class of single crystals featuring a monolayer or multiple layers of organic molecules that are periodically assembled along two dimension direction via supramolecular interactions such as hydrogen bonds, *π*–*π* stacking, and van der Waals forces.^[^ ^1^, ^2^, ^3^, ^4^, ^5^, ^6^, ^7^, ^8^, ^9^, ^10^, ^11^ ^]^ In recent years, they have attracted more and more attention in developing new‐generation supramolecular electronics owing to their unique physical, chemical, and mechanical properties, such as restricted carrier accumulation and transport, and polaronic relaxation, compared with their bulk counterparts.^[^ ^12^, ^13^, ^14^, ^15^ ^]^ In particular, novel phenomena have been also identified in 2DOMCs, including breaking the Landauer limit in a molecular diode, Peltier cooling in molecular junctions, molecular spinterfaces, and humidity‐controlled rectification switching.^[^ ^16^, ^17^, ^18^, ^19^ ^]^ Moreover, building blocks for 2DOMCs are organic molecules and have better solubility in various solvents under mild environments^[^ ^1^, ^2^, ^3^, ^4^, ^5^, ^6^, ^7^, ^8^, ^9^, ^10^, ^20^ ^]^ than ones of inorganic crystals (e.g., graphene, black phosphorus, Sb_2_O_3_, etc.),^[^ ^21^, ^22^, ^23^ ^]^ which is in favor of 2D organic crystallization by using the solution method. In particular, organic molecules are easily designed and tailored to be the ones with specific functional structures for new functional molecular crystals.^[^ ^24^, ^25^, ^26^, ^27^, ^28^, ^29^, ^30^ ^]^ Especially those 2DOMCs derived from *π*‐conjugated compounds exhibit potentially excellent electron‐accepting or donating and electron‐transporting properties under light irradiation and play a critically important role in the field of optoelectronic applications.

As is well‐known, the solution‐based approach is more practical for organic crystallization and it takes advantage of the solution processability of organic molecules and enables them to assemble into the corresponding 2DOMCs.^[^ ^1^, ^2^, ^3^, ^4^, ^5^, ^6^, ^7^, ^8^, ^9^, ^10^ ^]^ However, polycrystalline 2DOMCs with limited domain sizes are commonly observed in this strategy owing to uncontrolled crystallization speed during rapid solvent evaporation in open systems and random Brownian motion of the solute or solvent molecules under uncontrolled supersaturation in the solution state. This generally leads to the formation of voids, defects, and grain boundaries in the formed crystals.^[^ ^2^ ^]^ Moreover, the template is necessary for growth of 2DOMCs in the solution, which determines that possible template effects which are disadvantageous to the growth of 2D molecular crystal cannot be avoided. Meanwhile, planar *π*‐conjugated organic molecules with strong *π*–*π* stacking is easier to form higher crystalline 2DOMCs in the solution process, but it is still difficult to eliminate these inherent shortcomings of solution crystallization methods. In particular, the neat crystallization of 0D or cage‐like molecules becomes more difficult under similar conditions.^[^ ^1^, ^2^, ^3^, ^4^, ^5^, ^6^, ^7^, ^8^, ^9^, ^10^ ^]^ This is mainly attributed to two reasons: i) the contact areas among 0D molecules are much smaller than those of planar *π*‐conjugated molecules, resulting in weaker driving forces during their crystallization; ii) 0D molecules are easier to rotate on surfaces or interfaces without a preferential orientation.^[^ ^31^ ^]^ In fact, no matter if planar or cage‐like molecules are used, the difficulties encountered in solution crystallization method generally have negative effects on the quality of the resulting crystals. Due to randomness of material supply and mass transfer in the solution state, the growth of large‐area 2D single crystals becomes more difficult.^[^ ^31^ ^]^ It should be emphasized that highly efficient synthesis of large‐area and high‐quality 2DOMCs is crucial for complying with the integration and miniaturization trends in the electronic industry. Therefore, to develop an efficient and universal strategy for the growth of large‐area 2DOMCs in a controlled manner remains significant and challenging for real applications, e.g. photodetection.^[^ ^32^, ^33^ ^]^

As a newly developed crystallization medium,^[^ ^34^, ^35^, ^36^, ^37^, ^38^, ^39^ ^]^ supramolecular gel combines spatial confinements of 3D networks and strong or weak coupling with compounds to be crystallized. It exhibits reversible gelator‐assembled 3D networks within which solvent molecules and solutes are entrapped with restricted flowability. In this regard, the solid‐like gel phase entrapping bulk solvents greatly reduces the influence of solvent convection and external vibration on nucleation. Meanwhile, the gel networks suppress crystal sedimentation and aggregation at the solid/liquid interface, and inhibit positively the arbitrary crystallization along faces in contact with the vial walls or the early formed crystals. In this point, the similar phenomenon of geometrically‐confined lateral 2D crystal growth can be seen in a facile roll‐printing method for the preparation of large‐scale, single‐crystal CH_3_NH_3_PbI_3_ perovskite thin films. The geometrical confinement leads to the unidirectional lateral growth of single‐crystals on the instant crystallization from the perovskite feeding solution,^[^ ^40^ ^]^ which confirms that 3D networks of supramolecular gel will to a large extent provide confined spaces for 2D molecular crystallization. More importantly, control of supramolecular balances by introducing activated sites into gel networks can decide whether homogenous or heterogeneous nucleation is dominated during crystallization. Motion or dispersion of solvent or anti‐solvent, and solute within the gel phase can be modulated by changing the concentration, gel density, and environmental temperature.^[^ ^41^, ^42^ ^]^ To summarise, supramolecular gel can provide an activated medium like “parent crystals” to give birth to “daughter crystals” within confined spaces of the gel phases like co‐assembly^[^ ^43^ ^]^ and polymer‐induced crystallization.^[^ ^44^ ^]^ The resulting crystals can be separated from the gel–crystal mixtures by using some chemical or physical stimulus. Therefore, supramolecular gel crystallization (SGC) will be potentially a new strategy to grow neat 2DOMCs.

**Figure 1:**
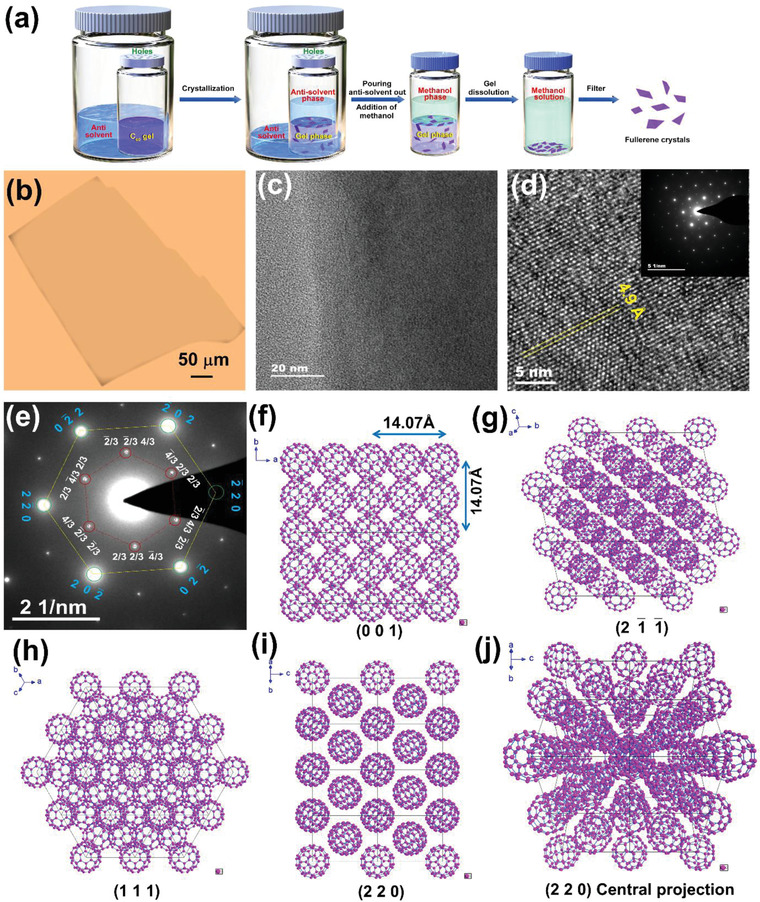
a) Preparation procedure for large‐area 2D fullerene molecular crystals; b) Optical image; c) TEM image, d) HR‐TEM image, and e) SAED images of 2D fullerene molecular crystals; f–j) structures of fullerene single crystals on various crystal planes.

Herein, we for the first time proposed and realized the growth of large‐area 2D molecular crystals using the SGC strategy with the assistance of anti‐solvent vapor diffusion. Experimentally, a small vial containing the gel containing compound to be crystallized was sealed with a sheet of aluminum foil with holes and placed in a larger vial containing the anti‐solvent. Then, the larger vial was sealed for a period to achieve complete crystallization. During this crystallization procedure, nucleation rates can be modulated by changing density of gel networks, concentration of compounds to be crystallized, antisolvent and environmental temperature, etc. To develop a general method for the growth of large‐area 2DOMCs, we chose a typical cage‐like organic semiconductor (C_60_) as a model molecule (**Figure** **1**a). Our considerations start from two points: i) Fullerene molecules usually tend to spontaneously self‐assembly into 1D crystals rather than 2D crystals in solution or gel phases except for the formation of nano‐sized peso‐2D fullerene co‐crystals with the help of specific templates.^[^ ^45^, ^46^, ^47^, ^48^, ^49^, ^50^ ^]^ In particular, fullerene is a typical 0D molecule and easy to move or rotate even on the high‐energy surface, which makes its growth into large‐area 2D single crystals in the solution state become more difficult than ones of planar molecules; ii) the fullerene molecule exhibits a conjugated structure consisting of sp^2^‐hybridized carbon atoms, and has been considered as a typical organic semiconductor in the fabrication of photoelectronic devices.^[^ ^51^, ^52^, ^53^, ^54^, ^55^, ^56^ ^]^ As far as we know, 2DOMC‐based optoelectronic devices derived from organic semiconductors commonly exhibit superior photoelectronic performances including high photosensitivity or high detectivity.^[^ ^2^ ^]^ Meanwhile, the SGC strategy has been proved in this work to be an efficient way to control fullerene to assemble into large‐area 2D single crystals for high‐performance photodetectors. Therefore, the SGC strategy is expected to be extended to provide a universal approach to grow more large‐area and high‐quality 2DOMCs for their potential applications.

## 2 Results and Discussion

**Scheme 1:**
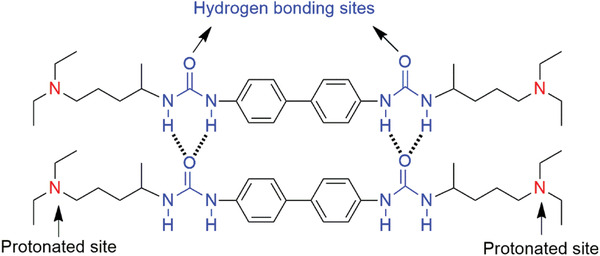
Molecular structure of **1** and its assembly mode via hydrogen bonding

In this study, we chose supramolecular gels derived from a bis(urea) bisurea‐based low‐molecular‐mass gelator bearing two diethylaminopentane moieties (1, **Scheme** **1**) developed previously^[^ ^41^, ^42^ ^]^ as media for the growth of 2D fullerene crystals. The main reasons for the choice in this work are i) that the used gelator with two urea fragments can efficiently gelate common aromatic solvents dissolving fullerene (Table S1, Supporting Information) into transparent gels (Figure S1, Supporting Information) within which organic crystallization can be monitored in situ; ii) that the resulting gels could maintain their stability well during dispersion of some anti‐solvent of fullerene, ensuring the gel media keeping very stable (Figure S2, Supporting Information); iii) that the gels can be turned into the clear solutions by the addition of some anti‐solvents of fullerene like methanol or acid (Figure S2, Supporting Information), providing a simple and efficient way to separate fullerene crystals from the gel–crystal mixture. Therefore, in this study, the mass transport or motion of fullerene and solvent molecules was tuned by varying the gelator concentration at a certain temperature. Acetonitrile was chosen as a model antisolvent of fullerene miscible with xylenes and diffused into the supramolecular gel phase in the vapor state to trigger fullerene supersaturation (Figure S3, Supporting Information). As a result, large‐area fullerene 2DOMCs in the gel phase formed within one week at a certain temperature (Figure 1b). As a poor solvent of fullerene, methanol can dissolve the gelator; therefore, it was used to induce gel–sol phase transition for the total liberation of the grown crystals from the gel media (Figure S3, Supporting Information). Of course, some additives that can strongly interact with gelator molecules, e.g. organic carboxylic acids or their salts, can also dissolve the urea gels for gel–crystal separation.^[^ ^34^, ^35^, ^41^ ^]^ Optical microscopy (OM), scanning electron microscopy (SEM), and atomic force microscopy examinations showed that assuming a rectangular shape the maximum 2DOMC area could reach ≈ 1.0 × 10^5^ µm^2^, which is the largest area yet reported,^[^ ^32^, ^33^, ^45^, ^46^, ^47^, ^48^, ^49^ ^]^ and the minimum thickness was commonly ≈3–5 nm (Figure 1b; Figure S4, Supporting Information).

**Figure 2:**
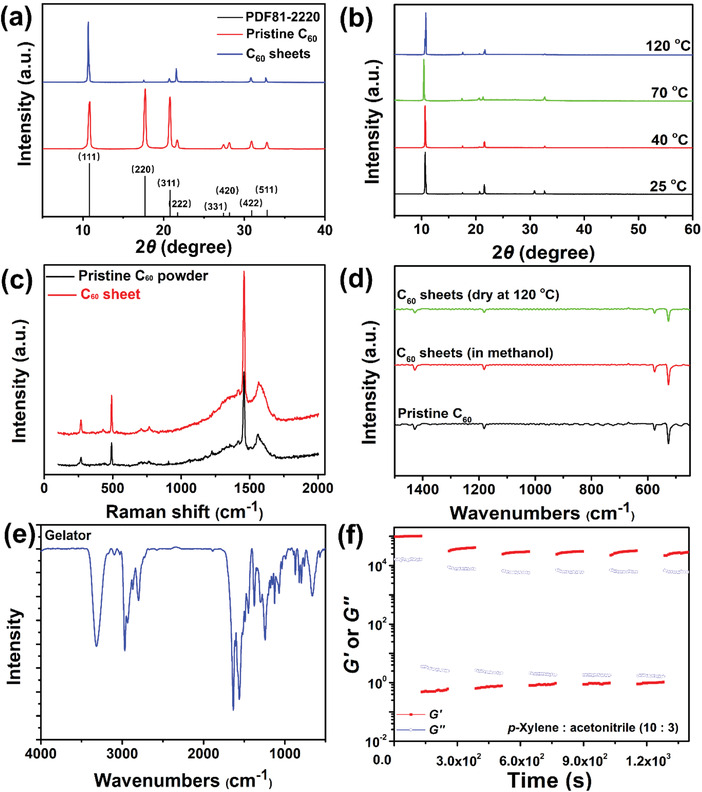
a) XRD patterns at room temperature of the 2D fullerene crystal and pristine fullerene powder; b) XRD signals of the 2D fullerene crystal during temperature quenching; c) Raman spectra of a fullerene sheet and fullerene raw powder; d) IR spectra of 2D fullerene crystals in the fresh state and dried state, compared with pristine fullerene powder; e) IR spectrum of the gelator; f) thixotropic behavior of the *p*‐xylene gel derived from the gelator.

High‐resolution transmission electron microscopy (HR‐TEM) and selected area electron diffraction (SAED) showed that the fullerene molecules assembled into 2D single crystals (Figure 1c,d). The lattice spacing between two (0 2 $\bar{2}$) crystal planes in the fullerene 2DOMC was 4.9 Å, consistent with powder X‐ray diffraction (XRD) measurements (insets in Figure 1d,e). The 2D crystals were not regular sheets, indicating that fullerene formed 2D single crystals along the {2 2 0} planes. In addition, the single‐crystal X‐ray diffraction (SCXRD) results showed that the 2D fullerene molecular crystal exhibited a face‐centred cubic (*fcc*) crystalline structure (a = b = c = 1.407 nm, Figure 1f–g; Table S2, Supporting Information).^[^ ^31^, ^42^, ^57^, ^58^ ^]^ The direction along the (111) crystal plane enhanced the thickness of the crystal (Figure 1h). A superlattice was observed in the SAED pattern (Figure 1e), which was in accordance with crystal planes such as (4/3 $\bar{2}$/3 $\bar{2}$/3) belonging to {2 $\bar{1}$ $\bar{1}$}. The XRD signals for the 2D fullerene crystal were identical to those of the pristine fullerene powder in the crystal form, exhibiting an *fcc* configuration (**Figure** **2**a). Moreover, the XRD spectra were unchanged with high‐temperature quenching from room temperature to 40, 70, and 120 °C under vacuum for 4 h per temperature (Figure 2b), showing that the formed crystals were very stable without any crystal change during thermal quenching. Infrared (IR) and Raman spectroscopy further confirmed that the resulting 2D fullerene crystals were very pure without any inserted impurities, including anti‐solvent, gelator, or gelated solvent (Figure 2c–e). These phenomena revealed that during 2D fullerene crystallization in the supramolecular gel, the gel fibres were not so strong as to insert into the formed crystals. In other words, the fullerene crystallization pressure was much higher than the gel stiffness.^[^ ^59^, ^60^ ^]^ Meanwhile, the gelated solvent also do not participate in cocrystallization with the fullerene molecules, which could be a critical reason for the high thermo‐stability of the resulting crystals. In fact, once a solvent participates in cocrystallization, the resulting crystal will transform into a new crystal form during thermal treatment.^[^ ^42^ ^]^

To clarify the key factors affecting fullerene crystallization in a supramolecular gel medium, the fullerene concentration and crystallization temperature were investigated for *p*‐xylene fullerene gels with acetonitrile vapor diffusion. With an increase in the fullerene concentration in the gel phase, fullerene grew from thinner 2DOMCs to irregular crystals at a certain crystallization temperature (Figure S5b,e,h,k,n, Supporting Information). Clearly, a higher fullerene concentration induced rapid crystallization with more nucleation sites in the gel phase, and aggregated crystals formed with a higher probability. Similarly, a temperature that was too low or too high was not conducive to neat 2D crystallization. This is understandable, as a higher crystallization temperature will induce more vigorous thermal motion of the fullerene and solvents within the gel networks, resulting in unstable nucleation and formation of irregular sheet‐like crystals or small crystal pieces (Figure S5c,f,i,l,o, Supporting Information).^[^ ^42^, ^46^ ^]^ Conversely, a lower crystallization temperature slows the thermal motion, and the fullerene molecules will move slowly within confined spaces of the gel networks (Figure S5a,d,g,j,m, Supporting Information), which prevents transport of the fullerene toward the 2D crystals within networks. In this case, the fullerene molecules commonly assemble into crystal blocks. Therefore, neat 2DOMCs can be formed only at a suitable temperature. Furthermore, the gelator concentration should not be ignored for controlling large‐area fullerene crystallization. In general, the density of the gel network changes with various gelator concentrations, which modulates the mass transport within the gel phases. Higher‐density networks inhibit the free motion of fullerene molecules,^[^ ^42^, ^46^ ^]^ ensuring that fullerene molecules are more likely to have time to assemble into neat 2DOMCs (Figure S6, Supporting Information). Conversely, the formation of fullerene nuclei becomes more random in relatively sparse gel networks at lower gelator concentrations. According to 2D crystal morphologies (Figures S5 and S6, Supporting Information), it can be clearly seen that the order of the impact on 2D fullerene crystallization in supramolecular gel is crystallization temperature > fullerene concentration > gelator concentration. During 2D crystallization, enhancement of the temperature or fullerene concentration would result in rapid motivation of fullerene molecules and solvent molecules. Conversely, lowering crystallization temperature, reducing fullerene concentration, or increasing gelator concentration will inhibit solute transport and nucleation rate in supramolecular gel. No matter which condition changes, crystallization kinetics is strongly dependent on mass transfer rate, deciding whether a neat crystal forms or not. This is also the bond of mutual relationship of these factors affecting crystallization together. As an optimized condition for the growth of neat 2D fullerene crystals, the crystallization was performed at 293 K (20 °C) by using a *p*‐xylene gel containing 1.0% (*w*/*v*) of the gelator and 0.5 mg mL^−1^ of fullerene under a vapor dispersion of 3 mL of acetonitrile in a closed space.

In the SGC strategy with the assistance of anti‐solvent vapor dispersion, the anti‐solvent and gelated solvent were shown to be important for 2D fullerene crystallization.^[^ ^37^, ^38^, ^39^, ^42^ ^]^ In this case, acetonitrile was chosen as the best anti‐solvent because it had the least effect on the gel networks during its vapor diffusion into the molecular gel phase at room temperature, unlike other anti‐solvents such as alcohols, THF, or acetone.^[^ ^42^ ^]^ The results show that solvent molecules with C_3_ and 6_3_ symmetries, such as *m*‐xylene and 1,3,5‐trimethylbenzene could fit into the C_3_‐symmetric cavity of the fullerene crystal spaces and commonly formed 1D fullerene crystals with a hexagonal close packed (*hcp*) structure in the solution or the gel state.^[^ ^42^, ^61^ ^]^ It was expected that 2D fullerene molecular crystals would be formed; therefore, two *meta* substituents in the structures of benzene derivatives were excluded in selecting the gelated solvents. To clarify the function of the gelated solvent in 2D crystallization, five commercially available aromatic solvents with two *para‐* or *ortho*‐substituent groups (*o*‐xylene, *p*‐xylene, 1,4‐diethylbenzene, 4‐ethyltoluene, and 4‐tert‐butyltoluene) were chosen to explore the effects of the gelated solvent on fullerene crystallization (Table S1, Supporting Information). The results showed that only *p*‐xylene gels could grow 2DOMCs, and massive crystals were formed in the other solvents (Figure S7, Supporting Information). To determine whether solvate crystals formed in the selected solvents, the IR spectra in Figure 2 and Figure S8 (Supporting Information) were obtained, showing that 1,4‐diethyl benzene and 4‐ethytolunene could insert into the spaces of the crystals, but *o*‐xylene, *p*‐xylene, and 4‐tert‐butyltoluene could not. XRD measurements showed that the crystals of *o*‐xylene, *p*‐xylene, 4‐ethytolunene, and 4‐tert‐butyltoluene exhibited face‐centred cubic (*fcc*) structures, while that of 1,4‐diethyl benzene exhibited an *hcp* structure (Figure S8, Supporting Information). Clearly, whether the gelated solvent participated in cocrystallization could not determine whether the crystallization would favor 1D, 2D, or 3D crystals.^[^ ^42^ ^]^ Furthermore, the energy‐minimized structures of the gelated solvents were estimated and are shown as insets in the corresponding SEM images, indicating that the *p*‐xylene molecule exhibited the highest symmetry. The differences in the subsequent morphologies of the fullerene crystals implied that more spatially symmetric solvents had a stronger tendency to form sheet‐like crystals. This could be mainly attributed to the strong interactions between *p*‐xylene and fullerene molecules, which was also supported by the corresponding solubility of fullerene in the solvents (Table S1, Supporting Information).

To further investigate why the SGC approach could produce large‐area 2D fullerene crystals, solution crystallization was carried out as a control experiment. In the solution method, anti‐solvent evaporation‐induced supersaturation produced irregular massive crystals (Figure S9a, Supporting Information), whereas in the fullerene gel state under suitable conditions, neat 2DOMCs were formed as designed (Figure S9c, Supporting Information). In particular, fullerene crystals obtained by the gel method exhibited higher‐quality crystallinity than those obtained by the solution method (Figure S9b,d, Supporting Information). As discussed in the section on solvent effects, *m*‐xylene or mesitylene commonly formed 1D fullerene crystals regardless of the solution or gel state, and fullerene crystals grew along a certain orientation driven by the forces from the cocrystallization of solvent molecules and fullerene molecules.^[^ ^42^, ^61^ ^]^ In *p*‐xylene, a more symmetric solvent, it was difficult for the solvent molecules to insert into the cavities of the fullerene crystal spaces during crystallization, which enhanced the possibility for the formation of fullerene crystals along various orientations.^[^ ^61^ ^]^ Therefore, the morphologies of fullerene crystals were more irregular in the solution method. Under these conditions, the growth of crystals along certain orientations at the lowest energy was inconspicuous, and isotropic crystallization was dominant. Only when this crystallization was spatially confined within some spaces like gel networks was the free movement of fullerene or solvent molecules for the casual growth of crystals strongly inhibited. Although the bisurea gelator with an aromatic spacer used in this study exhibited weak coupling with fullerene molecules in the gel state, as confirmed by rheology investigations (Figure S10, Supporting Information), the confined spaces of the supramolecular gel enhanced the specific attachment of fullerene onto some crystal planes with a slow dispersion velocity. In fact, the molecular packing is different for various crystal planes of the fullerene crystal, which will determine the degree of fullerene assembly onto these crystal planes. Within the gel network, the difference in the degree of formation along various crystal planes is enhanced, unlike in the solution state. As a result, crystallization was inclined toward an anisotropic state along some crystal planes on which fullerene molecules could easily assemble. It should be noted that the supramolecular gel also exhibited thixotropic behavior that the sol–gel transition can be reversible during applying or removing shear stress. That is to say, the gel networks or gel stiffness can recover partially or in whole upon removing shearing even though the anti‐solvent vapor diffused into the gel phase (Figure 2f).^[^ ^41^, ^42^ ^]^ This is another critical reason why this gel was selected to grow 2D fullerene molecular crystals. During 2D fullerene crystallization in the gel, the growing fullerene crystals with higher crystallization pressure pushed a portion of the supramolecular gel phase aside, and the resulting gel phase was self‐adaptive in terms of its thixotropic property at the same time. This ensured that the gel phase was stable during fullerene crystallization and prevented fullerene nuclei from casually assembling into irregular bulk crystals.

**Figure 3:**
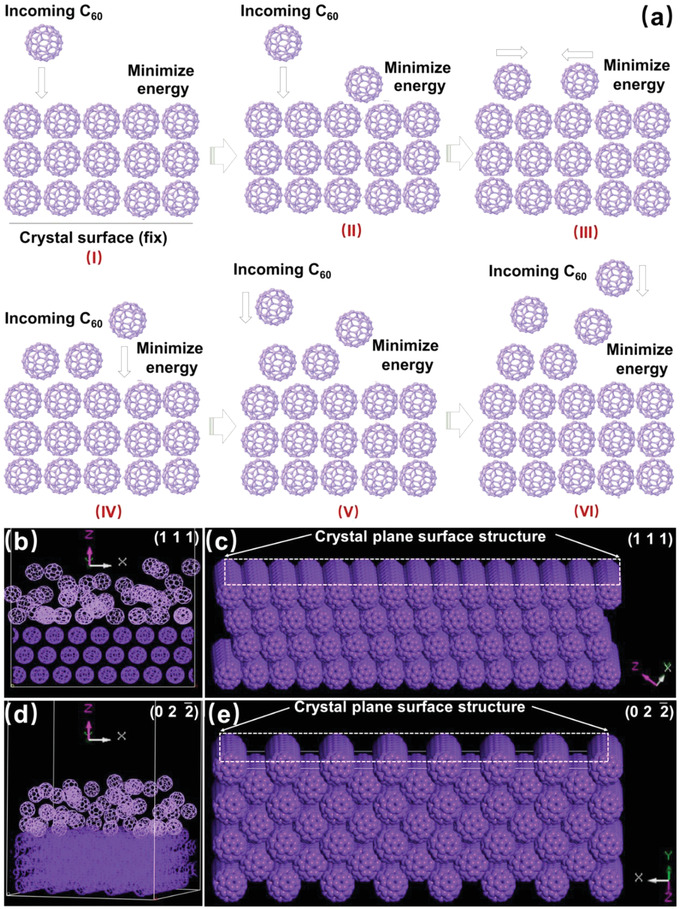
a) Diagram of the MDS process in which steps I–VI are repeated until 50 fullerene molecules are added above the crystal plate; optimized states of the b) (1 1 1) and d) (0 2 $\bar{2}$) crystal planes after gradual proliferation of 50 fullerene molecules, showing that the (0 2 $\bar{2}$) crystal plane is more dominant than the (1 1 1) crystal plane during slow crystallization; crystal plane surface structures of the c) (1 1 1) and e) (0 2 $\bar{2}$) crystal planes.

To confirm the role of the supramolecular gel, molecular dynamics simulations (MDS) were performed, as shown in **Figure** **3**. In this procedure, the (111) and (0 2 $\bar{2}$) planes were cleaved from the bulk single crystal as separate crystal planes. As shown in Figure 3a, fullerene molecules were released slowly one‐by‐one to mimic their behavior in the supramolecular gel phase. Procedure I‐VI are repeated until 50 C_60_ were deposited on the crystal surface. During this procedure, the interaction energy is calculated by using Universal Force field (Equation 1) and only translation and rotation of incoming C_60_ are considered (the configuration of C_60_ is rigid). The results revealed that fullerene molecules are more likely to form crystals on the (0 2 $\bar{2}$) crystal plane than on the (1 1 1) plane (Figure 3b,d). This is closely related to the structures of the two crystal planes. As shown in Figure 3c,e, the (0 2 $\bar{2}$) crystal plane exhibits more concave sites than the (1 1 1) crystal plane, which is favorable for the molecular assembly of fullerene as a highly rotational molecule. To further clarify the function of the gel during 2D fullerene crystallization, we placed the (0 2 $\bar{2}$) crystal plane (as a typical case) into a cell containing fullerene solution with gelator molecules, anti‐solvent, and gelated solvents. After mixing all the components to form a co‐gel onto the crystal plane, a three‐step mixing process was performed to observe the distribution of fullerene molecules on the crystal plane after the removal of gelators and solvents (Figure S11, Supporting Information). In addition, the gelator molecules in the co‐gel were removed from the remaining fullerene solution. The resulting solution was mixed gradually three times to examine the distribution of fullerene molecules on the crystal plane after the removal of all solvents (Figure S12, Supporting Information). The simulation results showed that the presence of the gelator molecules prevented fullerene molecules from moving rapidly onto the fullerene crystal plane. Rheological measurements of the co‐gel confirmed the existence of a weak coupling between the gelator and fullerene (Figure S10, Supporting Information). Therefore, it was concluded that within the gel networks the assembly of fullerene on the two crystal planes were discriminated by controlling mass transfer rate of fullerene molecules. As a result, the supramolecular gel mainly provides a spatially confined medium for controlling the molecular assembly of fullerene into 2DOMCs with face‐selected growth, overcoming the randomness of solution crystallization.

(1) $$U_{\mathrm{AB}}=\sum_{i=1}^{N_{A}}\sum_{j=1}^{N_{B}}u_{\mathrm{ij}}$$

Where, *u_ij_* is van der Waals interaction energy of atoms *i* and *j*.

For a more intuitive understanding of 2D crystallization, fresh gel samples were sandwiched and examined in parallel occasionally using OM. In this procedure, dispersion of the anti‐solvent into the *p*‐xylene gel at a certain temperature resulted in fullerene supersaturation, and an increasing number of nuclei gradually formed within the gel networks. Over time, an increasing number of fullerene molecules moved to facilitate the growth from nanocrystals to larger crystals (Figure S13, Supporting Information). In summary, fullerene crystallization could begin with a random nucleus within the 3D network under anti‐solvent‐triggered supersaturation within the confined spaces of the gel media. A schematic representation of the 2D crystallization is presented in Figure S14 (Supporting Information), and the SGC method exhibits typical superiority for growing large‐area fullerene 2DOMCs (Table S3, Supporting Information).

**Figure 4:**
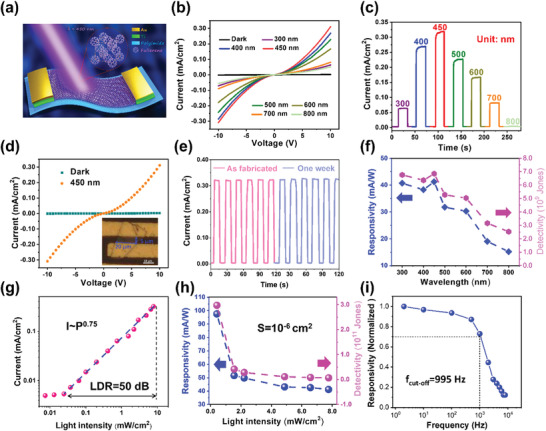
Photodetection performance of the flexible device derived from 2D fullerene single crystals: a) Schematic depiction of the flexible device; b) *I*–*V* measurements of the 2D C_60_ device with incident light of different wavelengths at the same illumination power of 7.76 mW cm^−2^; c) Time‐dependent photoresponse measurements of the C_60_ device with the light on and off at a voltage of 10 V and a wavelength of 450 nm; d) *I*–*V* measurements of the device with incident light of different wavelengths at the same illumination power of 7.76 mW cm^−2^, inset is the photograph of the flexible device based on a 2D fullerene molecular crystal; e) The comparative photodetecting performance of 2D C_60_ device under irradiation of 450 nm light after one week; f) The responsivity and detectivity of the 2D C_60_ device measured at different wavelength of light with various light intensities; g) The extracted current at different light intensities for the evaluation of linear dynamic range; h) The responsivity and detectivity of the 2D C_60_ device measured at different light intensities; i) The frequency‐dependent responsivity of the 2D C_60_ device under 450 nm irradiation.

Fullerene (C_60_) is a typical 0D organic semiconductor. In particular, its 2D molecular crystal has the potential to be one of the most widely used crystalline materials for supramolecular flexible electronics such as other 2D materials (e.g., organic field‐effect transistors and photodetectors)^[^ ^62^, ^63^, ^64^, ^65^, ^66^, ^67^, ^68^, ^69^ ^]^ because of its excellent optoelectronic properties, including its large light absorption coefficient.^[^ ^69^ ^]^ Therefore, the crystals released from the supramolecular gel by alcohol dissolution were transferred onto a flexible polyimide substrate to fabricate a flexible photoelectric device, as shown in Figure **4**a and the SEM image (inset in **Figure** **4**d). The voltage‐dependent current (*I*–*V*) curves of the 2DOMC flexible device were examined, as shown in Figure 4b in dark conditions and under irradiation at wavelengths of 300–800 nm. Figure 4c shows the time‐dependent current curves (*I*–*t*) for these wavelengths of incident light at a voltage of 10 V. The curves exhibited an initial increase and subsequent decrease with the increase in the wavelength of incident light from 300 to 800 nm, reaching a maximum at a wavelength of 450 nm. The on/off ratio was close to 100, and the response or decay time was less than 50 ms for 450 nm light (Figure 4d,e). The length and width of the device are about 20.0 and 5.0 µm, respectively, and the effective area is 10^−6^ cm^2^. We measure the stability of the device on aging in the air after one week. the photocurrent maintains unchanged after placing in the air for one week. Moreover, the response and decay speed also maintain unbated after one week, demonstrating the excellent response stability of our device. We also normalize intensity of photocurrent measured at different wavelengths (300–800 nm) as shown in Figure 4f according to the literature.^[^ ^70^ ^]^ The responsivity and detectivity of 2D‐C_60_ device are calculated to be as 41.2 mA W^−1^ and 2.9 × 10^11^ Jones at 10 V bias under 450 nm light, then start to decay when the wavelength is larger than 450 nm. This corresponds to the band gap of 2D fullerene crystal, about 1.91 eV (maximum absorption wavelength is ≈650 nm) (Figure S15, Supporting Information). Moreover, the photocurrent increases little when the light intensity is low, but inhibits quick rise when the light intensity is larger than 0.0245 mW cm^−2^ as shown in Figure S16 (Supporting Information), suggesting a close relationship between the photogeneration efficiency of the charge carriers and the absorbed photon flux. Figure 4g shows that the light intensity‐dependent photocurrent curve could be fitted with the power law *I*_p_∼*P* ^*θ*^, where *I*_p_ and *P* represent the photocurrent and the intensity of incident light, respectively. The linear dynamic range (LDR) is calculated as 50 dB. The corresponding exponent, *θ*, was calculated to be 0.75 (0.5 < *θ* < 1) for the fitting, showing the process of electron‐hole generation, separation, and trapping. As the light intensity increases, the responsivity and detectivity of 2D‐C_60_ device decrease, demonstrating an ability of detecting weak light (Figure 4h). We also measure the photoresponse performance of 2D‐C_60_ device with tunable frequency under irradiation of 450 nm light (Figure S17, Supporting Information). The photocurrent measurement at different frequency was shown in Figure 4i. The photocurrent starts to decay when the light frequency is higher than 100 Hz, and the cut‐off frequency could be calculated as 995 Hz.^[^ ^71^ ^]^ During the photo‐detection of the device, the 2D fullerene crystal generates electron–hole pairs under the illumination. The electron–hole pairs are further separated under external bias and arrive at the electrodes, during which photocurrent is generated.^[^ ^72^ ^]^

**Figure 5:**
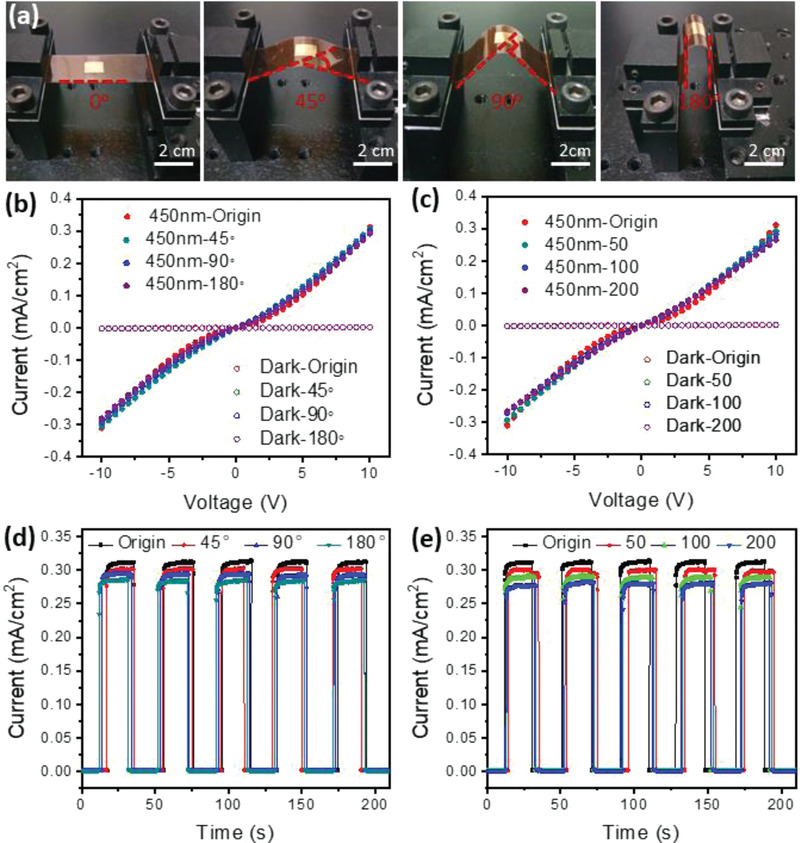
a) Photographs of the flexible device derived from 2D fullerene single crystals; b,c) Comparison of the *I*–*V* curves for the original sample and samples subjected to 50–200 bending cycles at 45 to 180° in the C_60_ device; d,e) Time‐dependent photoresponse measurements comparing the original sample and the samples subjected to 50–200 bending cycles at 45 to 180°.

More interestingly, the photocurrent and response speed remained unchanged after bending the flexible device at 45, 90, and 180° for 50, 100, and 200 cycles, as shown in **Figure** **5**. The contact also remained good even after 200 cycles of bending at various angles, as the *I*–*V* curve remained almost the same. To the best of our knowledge, this is the first case of a 2D fullerene crystal‐based flexible device, and the device in this study showed superior detectivity compared to previous results with pure molecular crystals, as summarised in Table S4 (Supporting Information). These results suggest that 2D fullerene crystals can be used to fabricate high‐quality, flexible photoelectric devices.

## 3 Conclusion

In summary, we developed a new methodology for growing 2DOMCs using the SGC strategy with the assistance of anti‐solvent vapor diffusion. When fullerene was chosen as a model molecule, the supramolecular gel provides spatial confinement with the help of a symmetric gelated solvent to control fullerene molecules and drive assembly selectively onto the {220} crystal plane. This determines whether fullerenes can be grown into 2DOMCs. The concentration of the gelator or fullerene and the crystallization temperature are critical for controlling the crystallization kinetics of the growth of neat 2D fullerene crystals. The resulting crystals exhibit high crystallinity, and the flexible device fabricated on a flexible substrate exhibits excellent bending and detection capacity performance. This study reveals that the gel plays a critical role in the growth of large‐area 2DOMCs and can promote the rapid development of SGC. This is expected to be an efficient and universal strategy to grow 2DOMCs of organic semiconductors for the fabrication of high‐performance devices.

## 4 Experimental Section

### Materials and Reagents

Fullerene, all solvents (e.g.*, o*‐xylene, *p*‐xylene, 1,4‐diethylbenzene, 4‐ethyltoluene, 4‐tert‐butyltoluene, acetonitrile, ethyl acetate, dichloromethane, and methanol), and all raw materials for synthesising the gelator were purchased from Japanese TCI Shanghai and used without further purification.

### Gelator Synthesis

The gelator was synthesised following the procedure reported previously^[^ ^41^, ^42^ ^]^ and characterised using standard methods, including ^1^H and ^13^C NMR (Bruker Avance 600 MHz NMR spectrometer) and high‐resolution mass spectrometry (Bruker Maxis UHR‐TOF Mass Spectrometer). 4,4’‐Diisocyanato‐3,3’‐dimethylbiphenyl and 4‐amino‐1‐diethyl‐aminopentane were dissolved at a 1:2 mass ratio in the proper sequence in dichloromethane with vigorous stirring in an ice bath, and then the mixture was stirred for ≈20 h at 25 °C under a nitrogen atmosphere. The resulting white precipitate was filtered and washed several times with ethyl acetate. Then, the washed precipitates were dried under vacuum and finally collected as white powder with a yield of 80%–90%.

### Fullerene Crystallization in Supramolecular Gels

A certain amount of fullerene was dissolved in a selected solvent under sonication as a brownish‐red solution. Then, the gelator powder was dissolved in the above solution under sonication and a warming and cooling cycle to form a brownish‐red transparent gel. The hybrid gel was placed in a small vial sealed with aluminum foil containing holes and placed in a larger vial containing a certain volume of anti‐solvent and sealed with a curved cap. The vials were transferred to an electro‐heating standing‐temperature cultivator at the set temperature, and 2D fullerene crystallization was allowed to proceed for approximately one week. The crystallization was monitored using OM at regular intervals. For experiments, a piece of fresh gel was removed from one of the smaller vials in parallel tests and sandwiched between two thinner glass slides sealed with the solvent to avoid further nucleation resulting from the evaporation of the gelated solvent.

### Fabrication of the 2D Fullerene Flexible Device

For device fabrication, a polyimide polymer with a thickness of 200 µm was chosen as the flexible substrate. The substrate was first cleaned under sonication using isopropanol, acetone, and alcohol in the proper sequence, and then dried under a flow of nitrogen gas. The 2D fullerene molecular crystals were dispersed in ethanol and then transferred onto the surface of the polyimide, followed by intensive drying under N_2_ gas. The standard photolithography technique (MDA‐400M, Midas) was followed by Cr/Au (10 nm/90 nm) electrode deposition with electron beam evaporation (Nexdep, Angstrom Engineering) using a standard lift‐off procedure. The channel area was ≈10^−6^ cm^2^. The 2D fullerene crystal used for device fabrication was cultivated in *p*‐xylene gel with the assistance of acetonitrile vapor diffusion ([Gelator] = 1.0% (*w*/*v*), [fullerene] = 0.5 g mL^−1^, 20 °C). The resulting crystal on the device exhibited an *fcc* structure growing along the {220} planes (as seen in the XRPD and SCXRD structures).

### Characterization Methods

A stress‐controlled rheometer (TA Company, USA, AR‐G2) was used to examine the rheological behavior of the hybrid gels in stress and time sweeps. Stress and time sweeps were performed with two steps per cycle to explore the changes in gel strength and thixotropic properties before and after anti‐solvent vapor diffusion into the hybrid gels. The morphology of the crystals was examined using an environmental scanning electron microscope (SEM, FEI, Quanta 200) with a sputter‐coating of Au. Structural characterization of the crystals was performed using a field transmission electron microscope (TEM, FEI, TecnaiG2 F20), X‐ray diffractometry (XRD, Rigaku, MiniFlex600), and single‐crystal X‐ray diffraction. FT‐IR spectroscopy (Bruker, Vertex 70 v) was used to examine whether the solvent or gelator was embedded in the spaces of the fullerene crystals. *I*–*V* and *I*–*t* measurements were recorded using a low‐temperature cryogenic probe station (Lake Shore, CRX‐6.5K), and semiconductor characteristic curves were recorded using a semiconductor parameter analyzer (Keithley, 4200‐SCS). A white light source (LDLS, Energetiq, EQ‐1500) provided monochromatic light with wavelengths ranging from 200 to 2400 nm. A UV‐enhanced Si photodiode was used to calibrate the light intensity.

## Conflict of Interest

The authors declare no conflict of interest.

## Supporting information

Supporting Information

## Data Availability

The data that support the findings of this study are available from the corresponding author upon reasonable request.

## References

[1] E. Ahmed , D. P. Karothu , M. Warren , P. Naumov , Nat. Commun. 2019, 10, 3723. 31427570 10.1038/s41467-019-11612-z PMC6700106

[2] B. B. Fu , C. Wang , Y. T. Sun , J. R. Yao , Y. Wang , F. Y. Ge , F. X. Yang , Z. Y. Liu , Y. F. Dang , X. T. Zhang , X. F. Shao , R. J. Li , W. P. Hu , Adv. Mater. 2019, 31, 1901437. 10.1002/adma.201901437 31268577

[3] J. R. Yao , Y. Zhang , X. Z. Tian , X. L. Zhang , H. J. Zhao , X. T. Zhang , J. S. Jie , X. R. Wang , R. J. Li , W. P. Hu , Angew. Chem., Int. Ed. Engl. 2019, 58, 16082. 31432576 10.1002/anie.201909552

[4] J. Qian , S. Jiang , S. L. Li , X. R. Wang , Y. Shi , Y. Li , Adv. Mater. Technol. 2019, 4, 1800182.

[5] S. K. Park , J. H. Kim , S. Y. Park , Adv. Mater. 2018, 30, 1704759.

[6] F. X. Yang , S. S. Cheng , X. T. Zhang , X. C. Ren , R. J. Li , H. L. Dong , W. P. Hu , Adv. Mater. 2018, 30, 1702415. 10.1002/adma.201702415 29024065

[7] C. L. Tan , X. H. Cao , X. J. Wu , Q. Y. He , J. Yang , X. Zhang , J. Z. Chen , W. Zhao , S. K. Han , G. H. Nam , M. Sindoro , H. Zhang , Chem. Rev. 2017, 117, 6225. 28306244 10.1021/acs.chemrev.6b00558

[8] Y. H. Zhang , J. S. Qiao , S. Gao , F. R. Hu , D. W. He , B. Wu , Z. Y. Yang , M. Xiao , H. X. Xu , J. B. Xu , X. R. Wang , Phys. Rev. Lett. 2016, 116, 016602. 26799035 10.1103/PhysRevLett.116.016602

[9] G. Fiori , F. Bonaccorso , G. Iannaccone , T. Palacios , D. Neumaier , A. Seabaugh , S. K. Banerjee , L. Colombo , Nat. Nanotech. 2014, 9, 768. 10.1038/nnano.2014.207 25286272

[10] N. R. Champness , Nat. Chem. 2014, 6, 757. 25143206 10.1038/nchem.2015

[11] L. X. Liu , T. Y. Zhai , InfoMat 2021, 3, 3.

[12] Y. Zhang , J. Qiao , S. Gao , F. Hu , D. He , B. Wu , Z. Yang , B. Xu , Y. Li , Y. Shi , W. Ji , P. Wang , X. Wang , M. Xiao , H. Xu , J. B. Xu , X. Wang , Phys. Rev. Lett. 2016, 116, 16602. 10.1103/PhysRevLett.116.016602 26799035

[13] X. Zhuang , Y. Mai , D. Wu , F. Zhang , X. Feng , Adv. Mater. 2015, 27, 403. 25155302 10.1002/adma.201401857

[14] S. L. Cai , W. G. Zhang , R. N. Zuckermann , Z. T. Li , X. Zhao , Y. Liu , Adv. Mater. 2015, 27, 5762. 25735971 10.1002/adma.201500124

[15] J. J. Brondijk , W. S. C. Roelofs , S. G. J. Mathijssen , A. Shehu , T. Cramer , F. Biscarini , P. W. M. Blom , D. M. D. Leeuw , Phys. Rev. Lett. 2012, 109, 056601. 23006192 10.1103/PhysRevLett.109.056601

[16] X. P. Chen , M. Roemer , L. Yuan , W. Du , D. Thompson , E. D. Barco , C. A. Nijhuis , Nat. Nanotech. 2017, 12, 797. 10.1038/nnano.2017.110 28674457

[17] L. J. Cui , R. J. Miao , K. Wang , D. Thompson , L. A. Zotti , J. C. Cuevas , E. Meyhofer , P. Reddy , Nat. Nanotech. 2018, 13, 122. 10.1038/s41565-017-0020-z 29255291

[18] Cinchetti, M. , Dediu, V. A. , Hueso, L. E. , Nat. Mater. 2017,16, 507. 28439116 10.1038/nmat4902

[19] H. Atesci , V. Kaliginedi , J. A. C. Gil , H. Ozawa , J. M. Thijssen , P. Broekmann , M. Haga , S. J. van der Molen , Nat. Nanotech. 2018, 13, 117. 10.1038/s41565-017-0016-8 29203913

[20] X. Zhang , C. H. Hsu , X. Ren , Y. Gu , B. Song , H. J. Sun , S. Yang , E. Chen , Y. Tu , X. Li , Angew. Chem., Int. Ed. Engl. 2015, 54, 114. 25327867 10.1002/anie.201408438

[21] K. S. Novoselov , A. Mishchenko , A. Carvalho , A. H. Castro Neto , Science 2016, 353, aac9439. 27471306 10.1126/science.aac9439

[22] W. Han , P. Huang , L. Li , F. K. Wang , P. Luo , K. L. Liu , X. Zhou , H. Q. Li , X. W. Zhang , Y. Cui , T. Y. Zhai , Nat. Commun. 2019, 10, 4728. 31624241 10.1038/s41467-019-12569-9 PMC6797790

[23] F. K. Wang , S. J. Yang , J. Wu , X. Z. Hu , Y. Li , H. Q. Li , X. T. Liu , J. H. Luo , T. Y. Zhai , InfoMat 2021, 3, 1251.

[24] Y. J. Shi , L. Jiang , J. Liu , Z. Y. Tu , Y. Y. Hu , Q. H. Wu , Y. P. Yi , E. Gann , C. R. McNeill , H. X. Li , W. P. Hu , D. B. Zhu , H. N. Sirringhaus , Nat. Commun. 2018, 9, 2933. 30050114 10.1038/s41467-018-05390-3 PMC6062560

[25] M. Cao , C. Zhang , Z. Cai , C. C. Xiao , X. S. Chen , K. Y. Yi , Y. G. Yang , Y. H. Lu , D. C. Wei , Nat. Commun. 2019, 10, 756. 30765699 10.1038/s41467-019-08573-8 PMC6375977

[26] H. Y. Li , Y. J. Shi , G. C. Han , J. Liu , J. Zhang , C. L. Li , J. Liu , Y. P. Yi , T. Li , X. K. Gao , C. G. Di , J. Huang , Y. K. Che , D. Wang , W. P. Hu , Y. Q. Liu , L. Jiang , Angew. Chem., Int. Ed. Engl. 2020, 59, 4380.31943644

[27] D. W. He , Y. H. Zhang , Q. S. Wu , R. Xu , H. Y. Nan , J. F. Liu , J. J. Yao , Z. L. Wang , S. J. Yuan , Y. Li , Y. Shi , J. L. Wang , Z. H. Ni , L. He , F. Miao , F. Q. Song , H. X. Xu , K. W. Taniguchi , J. B. Xu , X. R. Wang , Nat. Commun. 2014, 5, 5162. 25330787 10.1038/ncomms6162

[28] Y. L. Duan , S. Semin , P. Tinnemans , H. Cuppen , J. L. Xu , T. Rasing , Nat. Commun. 2014, 10, 4573. 10.1038/s41467-019-12601-y PMC6783412 31594954

[29] H. J. Zhao , Y. B. Zhao , Y. X. Song , M. Zhou , W. Lv , L. Tao , Y. Z. Feng , B. Y. Song , Y. Ma , J. Q. Zhang , J. Xiao , Y. Wang , D. H. Lien , M. Amani , H. J. Kim , X. Q. Chen , Z. T. Wu , Z. H. Ni , P. Wang , Y. Shi , H. B. Ma , X. Zhang , J. B. Xu , A. Troisi , A. Javey , X. R. Wang , Nat. Commun. 2019, 10, 5589. 31811122 10.1038/s41467-019-13581-9 PMC6897925

[30] Q. Wang , J. Qian , Y. Li , Y. Zhang , D. He , S. Jiang , Y. Wang , X. Wang , L. Pan , J. Wang , X. Wang , Z. Hu , H. Nan , Z. Ni , Y. Zheng , Y. Shi , Adv. Funct. Mater. 2016, 26, 3191.

[31] M. Li , K. Deng , S. B. Lei , Y. L. Yang , T. S. Wang , Y. T. Shen , C. R. Wang , Q. D. Zeng , C. Wang , Angew. Chem., Int. Ed. Engl. 2008, 47, 6717. 18655080 10.1002/anie.200802518

[32] M. Feng , J. Zhao , H. Petek , Science 2008, 320, 359. 18420931 10.1126/science.1155866

[33] M. Novak , A. Ebel , T. Meyer‐Friedrichsen , A. Jedaa , B. F. Vieweg , G. Yang , K. Voitchovsky , F. Stellacci , E. Spiecker , A. Hirsch , M. Halik , Nano Lett.. 2011, 11, 156. 21133354 10.1021/nl103200r

[34] J. A. Foster , M. O. M. Piepenbrock , G. O. Lloyd , N. Clarke , J. A. K. Howard , J. W. Steed , Nat. Chem. 2010, 2, 1037. 21107367 10.1038/nchem.859

[35] D. K. Kumar , J. W. Steed , Chem. Soc. Rev. 2014, 43, 2080. 23925539 10.1039/c3cs60224a

[36] J. Buendia , E. Matesanz , D. K. Smith , L. Sanchez , CrystEngComm 2015, 17, 8146.

[37] J. A. Foster , K. K. Damodaran , A. Maurin , G. M. Day , H. P. G. Thompson , G. J. Cameron , J. C. Bernalc , J. W. Steed , Chem. Sci. 2017, 8, 78. 28451150 10.1039/c6sc04126d PMC5304659

[38] L. A. Estroff , L. Addadi , S. Weiner , A. D. Hamilton , Org. Biomol. Chem. 2004, 2, 137. 14737673 10.1039/b309731e

[39] E. Asenath‐Smith , H. Y. Li , E. C. Keene , Z. W. Seh , L. A. Estroff , Adv. Funct. Mater. 2012, 22, 2891.

[40] L. Lee , J. M. Baek , K. S. Park , Y. E. Lee , N. K. Shrestha , M. M. Sung , Nat. Commun. 2017, 8, 15882. 28691697 10.1038/ncomms15882 PMC5508126

[41] S. Gao , J. Ma , S. S. Wang , Y. Wu , X. W. Fu , R. K. Marella , K. Q. Liu , Y. Fang , Langmuir 2016, 32, 12805. 27794610 10.1021/acs.langmuir.6b03375

[42] K. Q. Liu , S. Gao , Z. Zheng , X. L. Deng , S. Mukherjee , S. S. Wang , H. Xu , J. Q. Wang , J. F. Liu , T. Y. Zhai , Y. Fang , Adv. Mater. 2019, 31, 1808254. 10.1002/adma.201808254 30873680

[43] Q. Tang , G. P. Zhang , B. H. Jiang , D. Y. Ji , H. H. Kong , K. Riehemann , Q. M. Ji , H. Fuchs , SmartMat. 2021, 2, 109.

[44] A. V. Fulari , N. T. Duong , D. A. Nguyen , Y. Jo , S. Cho , D. Y. Kim , N. K. Shrestha , H. Kim , H. Im , Chem. Eng. J. 2022, 433, 133809.

[45] L. K. Shrestha , Q. M. Ji , T. Mori , K. Miyazawa , Y. Yamauchi , J. P. Hill , K. Ariga , Chem. Asian J. 2013, 8, 1662. 23589223 10.1002/asia.201300247

[46] K. Lee , B. Choi , I. J. Pante , M. V. Paley , X. J. Zhong , A. C. Crowther , J. S. Owen , X. Y. Zhu , X. Roy , Angew. Chem., Int. Ed. Engl. 2018, 57, 6125. 29603561 10.1002/anie.201800953

[47] D. L. Cui , M. Ebrahimi , F. Rosei , J. M. Macleod , J. Am. Chem. Soc. 2017, 139, 16732. 29072461 10.1021/jacs.7b08642

[48] A. V. Zotov , D. A. Olyanich , V. V. Mararov , T. V. Utas , L. V. Bondarenko , A. Y. Tupchaya , D. V. Gruznev , A. N. Mihalyuk , C. M. Wei , Y. L. Wang , A. A. Saranin , J. Chem. Phys. 2018, 149, 034702. 30037255 10.1063/1.5038790

[49] C. Park , H. J. Song , H. C. Choi , Chem. Commun. 2009, 32, 4803. 10.1039/b909888g 19652786

[50] C. G. Bezzu , L. A. Burt , C. J. McMonagle , S. A. Moggach , B. M. Kariuki , D. R. Allan , M. Warren , N. B. McKeown , Nat. Mater. 2019, 18, 740. 31086318 10.1038/s41563-019-0361-0

[51] K. Szendrei , F. Cordella , M. V. Kovalenko , M. B. Berl , G. Hesser , M. Yarema , D. Jarzab , O. V. Mikhnenko , A. Gocalinska , M. Saba , F. Quochi , A. Mura , G. Bongiovanni , P. W. M. Blom , W. Heiss , M. A. Loi , Adv. Mater. 2009, 21, 683.

[52] K. S. Nalwa , Y. K. Cai , A. L. Thoeming , J. Shinar , R. Shinar , S. Chaudhary , Adv. Mater. 2010, 22, 4157. 20803757 10.1002/adma.201000417

[53] L. K. Shrestha , R. G. Shrestha , Y. Yamauchi , J. P. Hill , T. Nishimura , K. Miyazawa , T. Kawai , S. Okada , K. Wakabayashi , K. Ariga , Angew. Chem., Int. Ed. Engl. 2015, 54, 951. 25425340 10.1002/anie.201408856

[54] Z. Tang , Z. F. Ma , A. Sánchez‐Díaz , S. Ullbrich , Y. Liu , B. Siegmund , A. Mischok , K. Leo , M. Campoy‐Quiles , W. W. Li , K. Vandewal , Adv. Mater. 2017, 29, 1702184. 10.1002/adma.201702184 28675522

[55] F. W. Guo , Z. G. Xiao , J. S. Huang , Adv. Opt. Mater. 2013, 1, 289.

[56] R. Saran , R. J. Curry , Small 2018, 14, 1703624. 10.1002/smll.201703624 29350479

[57] C. Larsen , H. R Barzegar , F. Nitze , T. Wåberg , L. Edman , Nanotechnology 2012, 23, 344015. 22885636 10.1088/0957-4484/23/34/344015

[58] R. R. Shrestha , L. K. Shrestha , A. H. Khan , G. S. Kumar , S. Acharya , K. Ariga , ACS Appl. Mater. Interfaces 2014, 6, 15597. 25136819 10.1021/am5046235

[59] J. Ren , M. S. Niu , X. Y. Guo , Y. J. Liu , X. Y. Yang , M. Chen , X. T. Hao , Y. Zhu , H. Z. Chen , H. Y. Li , J. Am. Chem. Soc. 2020, 142, 1630. 31893499 10.1021/jacs.9b13087

[60] H. Y. Li , H. L. L. Xin , D. A. Muller , L. A. Estroff , Science 2009, 326, 1244. 19965470 10.1126/science.1178583

[61] M. Rana , R. B. Reddy , B. B. Rath , U. K. Gautam , Angew. Chem., Int. Ed. Engl. 2014, 53, 13523. 25324209 10.1002/anie.201408981

[62] Y. R. Lim , W. Song , J. K. Han , Y. B. Lee , S. J. Kim , S. Myung , S. S. Lee , K. S. An , C. J. Choi , J. S. Lim , Adv. Mater. 2016, 28, 5025. 27119775 10.1002/adma.201600606

[63] Q. Wei , J. H. Chen , P. Ding , B. Shen , J. Yin , F. Xu , Y. D. Xia , Z. G. Liu , ACS Appl. Mater. Interfaces 2018, 10, 21527. 29847912 10.1021/acsami.8b02582

[64] J. Li , Z. X. Wang , Y. Wen , J. W. Chu , L. Yin , R. Q. Cheng , L. Lei , P. He , C. Jiang , L. P. Feng , Adv. Funct. Mater. 2018, 28, 1706437.

[65] J. C. Zhang , Y. C. Huang , Z. J. Tan , T. R. Li , Y. C. Zhang , K. C. Jia , L. Lin , L. Z. Sun , X. W. Chen , Z. Z. Li , C. W. Tan , J. X. Zhang , L. M. Zheng , Y. Wu , B. Deng , Z. L. Chen , Z. F. Liu , H. L. Peng , Adv. Mater. 2018, 30, 1803194.

[66] J. D. Yao , G. W. Yang , Small 2018, 14, 1704524.

[67] M. J. Dai , H. Y. Chen , F. K. Wang , Y. X. Hu , S. Wei , J. Zhang , Z. G. Wang , T. Y. Zhai , P. A. Hu , ACS Nano 2019, 13, 7291. 31188571 10.1021/acsnano.9b03278

[68] R. Y. Wang , F. Y. Zhou , L. Lv , S. S. Zhou , Y. W. Yu , F. W. Zhuge , H. Q. Li , L. Gan , T. Y. Zhai , CCS Chem. 2019, 1, 448.

[69] F. W. Guo , Z. G. Xiao , J. S. Huang , Adv. Opt. Mater. 2013, 1, 289.

[70] J. Lee , S. J. Ko , H. Lee , J. F. Huang , Z. Y. Zhu , M. Seifrid , J. Vollbrecht , V. V. Brus , A. Karki , H. B. Wang , K. Cho , T.‐Q. Nguyen , G. C. Bazan , ACS Energy Lett.. 2019, 4, 1401.

[71] J. F. Huang , J. Lee , J. Vollbrecht , V. V. Brus , A. L. Dixon , D. X. Cao , Z. Y. Zhu , Z. F. Du , H. B. Wang , K. Cho , G. C. Bazan , T. Q. Nguyen , Adv. Mater. 2020, 32, 1906027. 10.1002/adma.201906027 31714629

[72] K. J. Baeg , M. Binda , D. Natali , M. Caironi , Y. Y. Noh , Adv. Mater. 2013, 25, 4267. 23483718 10.1002/adma.201204979

