# Supplementary material for: Spatially Confined Face‐Selective Growth of Large‐Area 2D Organic Molecular Crystals in a Supramolecular Gel for Highly Efficient Flexible Photodetection
Source: Adv Sci (Weinh). 2022 Sep 1;9(30):2203662. doi: 10.1002/advs.202203662 (PMC9596823; doi:10.1002/advs.202203662)
Supplement: Supplementary file 1 — Supporting Information [file ADVS-9-2203662-s001.pdf]

ADVANCED SCIENCE
Open Access

Supporting Information

for *Adv. Sci.*, DOI 10.1002/advs.202203662

Spatially Confined Face-Selective Growth of Large-Area 2D Organic Molecular Crystals in a Supramolecular Gel for Highly Efficient Flexible Photodetection

Chaowen Shen, Pan Han, Zhi Zheng, Wenhe Jiang, Sheng Gao, Chunxia Hua, Cheng Lung Chen, Fan Xia, Tianyou Zhai, Kaiqiang Liu* and Yu Fang*

Supporting Information

Spatially Confined Face-Selective Growth of 2D Fullerene Molecular Crystals in a Supramolecular Gel for Highly Efficient Flexible Photodetection

Chaowen Shen, Pan Han, Zhi Zheng, Wenhe Jiang, Sheng Gao, Chunxia Hua, Cheng Lung Chen, Fan Xia, Tianyou Zhai, Kaiqiang Liu,* Yu Fang*

Calculation methods for device performance indexes

1. Device responsivity (R_λ)

$R_\lambda = I_{ph}/PS$..(1)

The photocurrent can be calculated as $I_{ph} = I_{light} - I_{dark}$, where I_{light} is the current under illumination, and I_{dark} is the current under dark conditions. In this equation, P is the optical power density impinging on the device, S is the illuminated area, and T is transmittance of C_{60} crystals.

In this study, P = 7.76 mW/cm^2, S = 100 μm^2, λ = 450 nm, I = 311 pA, and I_{dark} = 2.12 pA.

Calculated result: R_λ = 41.2 mA/W

2. Specific detectivity (D^*)

$D^* = R_\lambda/(2e \times I_{dark}/S)^{1/2}$..(2)

In this study, R_λ = 97.6 mA/W, I_{dark} = 2.12 pA, S = 100 μm^2, and the specific detectivity is calculated directly from the dark current: D^* = 2.9 × 10^{11} Jones at a 10 V bias.

Table S1 Solubility of fullerene in selected solvents at 20 °C

Solvent	Solubility
Toluene	2.80×10^{-3} g·mL^{-1}
o-Xylene	3.33×10^{-3} g·mL^{-1}
p-Xylene	5.00×10^{-3} g·mL^{-1}
1,4-Diethylbenzene	4.76×10^{-4} g·mL^{-1}
4-Ethyltoluene	2.38×10^{-3} g·mL^{-1}
4-tert-Butyltoluene	2.70×10^{-3} g·mL^{-1}

Ref.: *J. Phys. Chem.***1993**, *97*, 3379; *Nanoscale* **2018**, *10*, 8170.

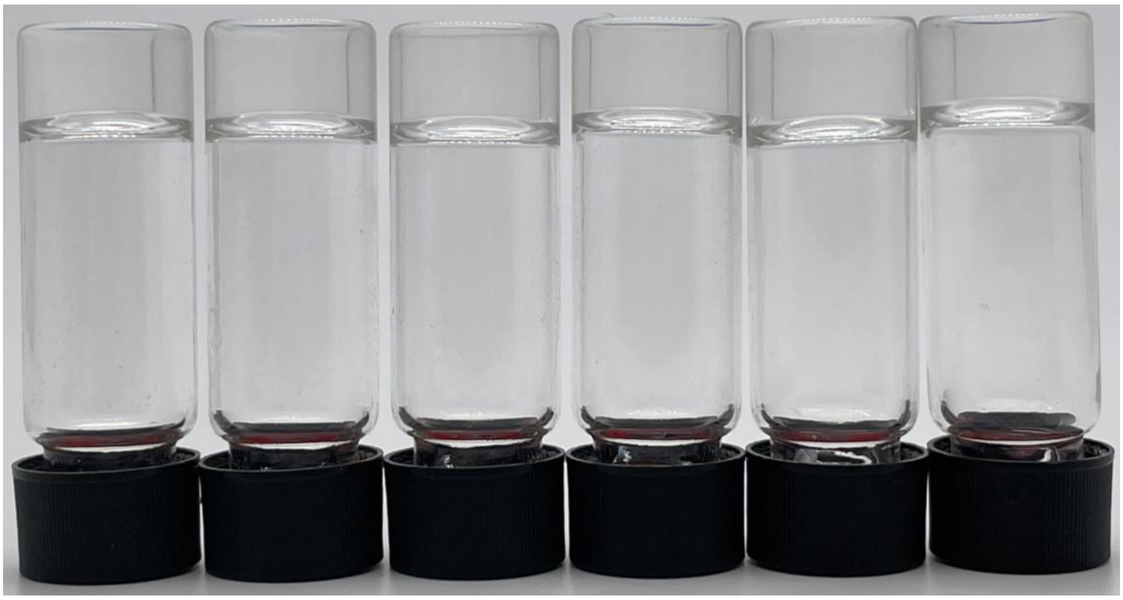

Figure S1. Transparent gels derived from the gelator **1** and aromatic solvents. From left to right in proper order: Toluene, *o*-xylene, *p*-xylene, *p*-diethylbenzene, 1-ethyl-4-methylbenzene, and *p*-tert-butyl toluene ([**1**] = 1% (w/v)).

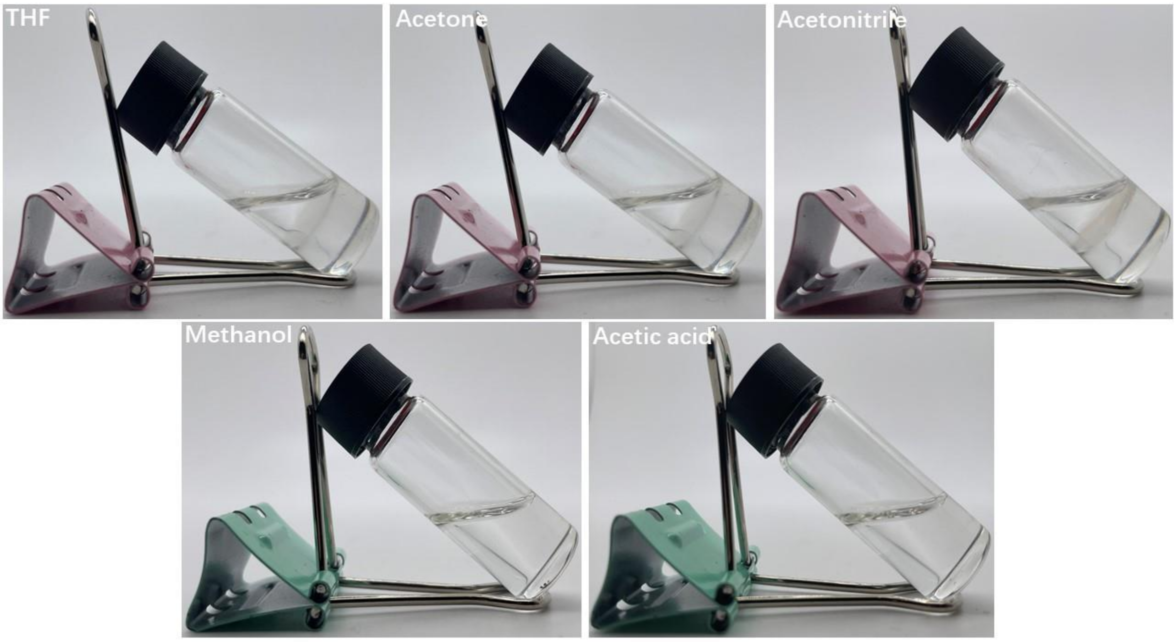

Figure S2. Tolerance of transparent gels (**1**/*p*-xylene, [**1**] = 1% (w/v)) towards antisolvents of fullerene (THF, acetone and acetonitrile); Dissolution of the gels by methanol or acetic acid.

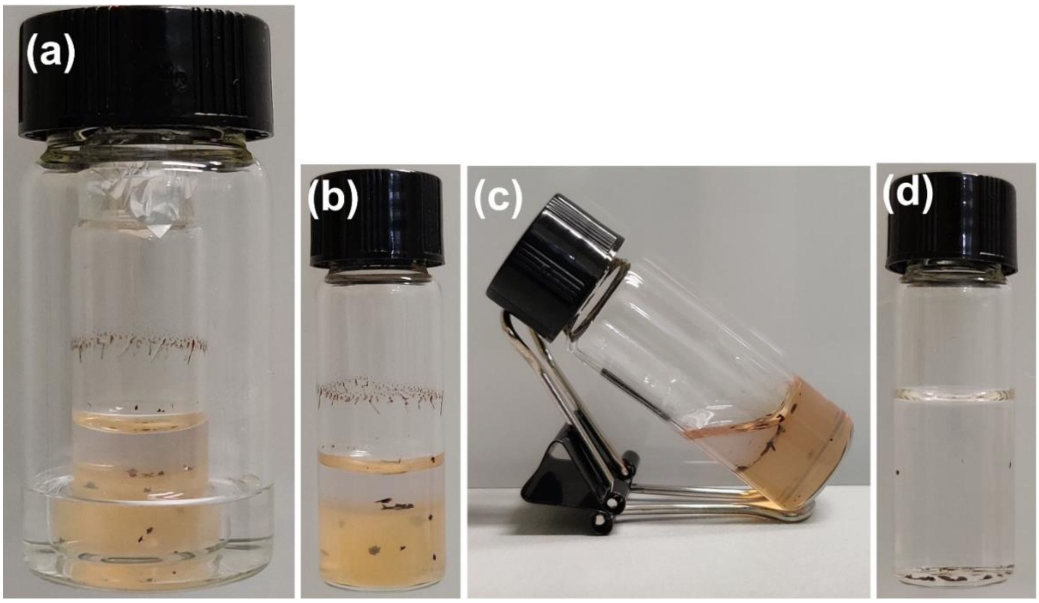

Figure S3. Preparation procedure and separation of large-size fullerene crystals using the supramolecular gel approach with evaporation of an anti-solvent: (a) crystallisation procedure; (b) small inner vial removed from the larger vial; (c) addition of methanol above the gel/crystal phase after the removal of the anti-solvent; (d) crystals in methanol after total dissolution of the gelator by methanol.

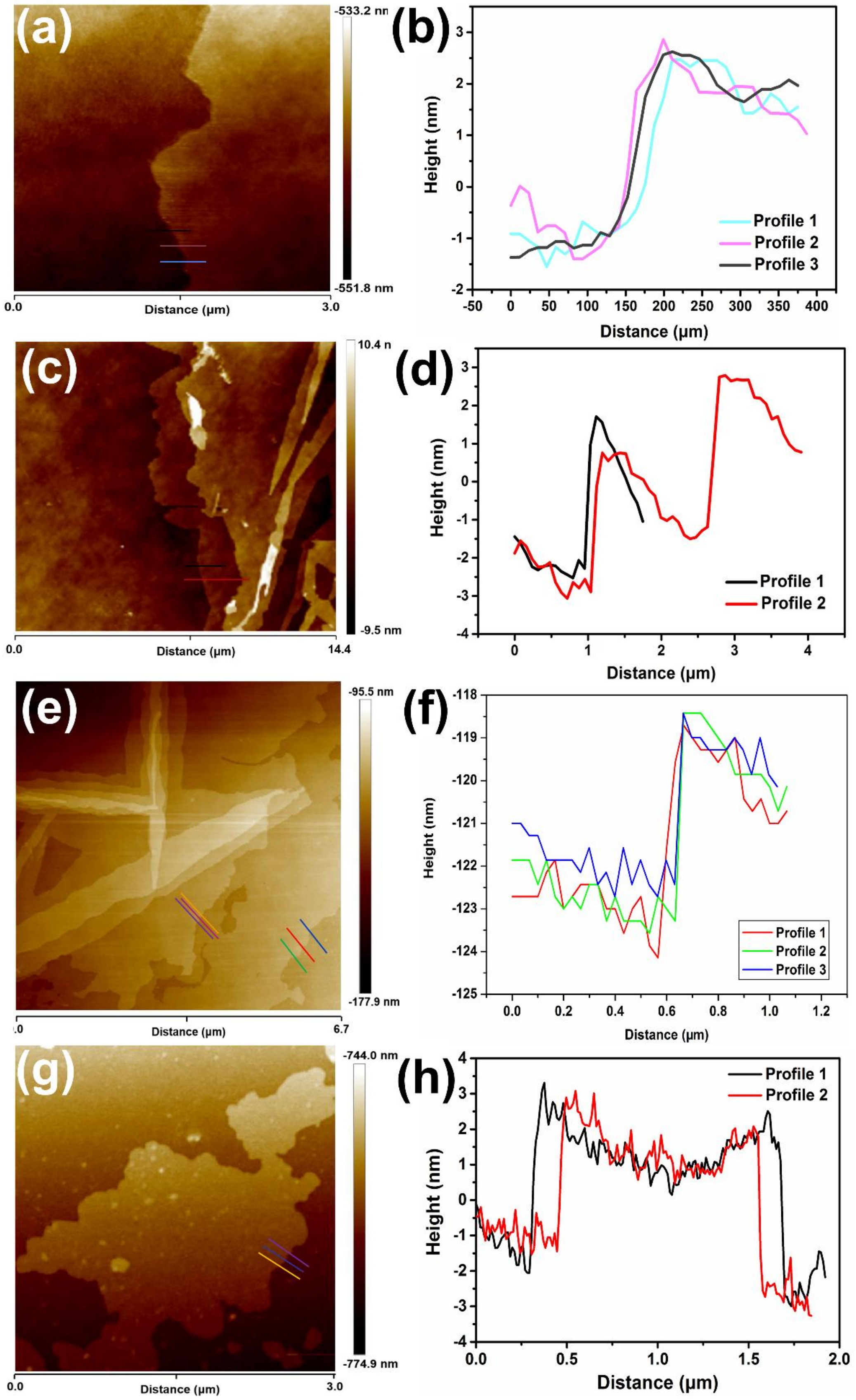

Figure S4. AFM images of C_{60} sheets growing in molecular gels derived from gelator **1** and *p*-xylene ([gelator] = 1% (w/v), [C_{60}] = 0.5 mg/mL).

Table S2. Crystal data and structure refinement for 2D C_{60}

Information	Details	
Identification code	2D C_{60}	
Empirical formula	C_{60}	
Formula weight	720.60	
Temperature	293(2) K	
Wavelength	1.54178 Å	
Crystal system	Cubic	
Space group	Pa -3	
Unit cell dimensions	a = 14.070 Å	α = 90°
	b = 14.070 Å	β = 90°
	c = 14.070 Å	γ = 90°
Volume	2785.4(2) $Å^3$	
Z	4	
Calculated density	1.718 mg/m^3	
Absorption coefficient	0.775 mm^{-1}	
F (000)	1440	
Crystal size	0.2 × 0.2 × 0.2 mm^3	
Theta range for data collection	5.445° to 72.203°	
Limiting indices	-12 ≤ h ≤ 12, -12 ≤ k ≤ 12, -17 ≤ l ≤ 17	
Reflections collected/unique	982/982 [R(int) = 0.1312]	
Completeness to theta = 67.679°	100.0%	
Refinement method	Full-matrix least-squares on F^2	
Data/restraints/parameters	982/91/95	
Goodness-of-fit on F2	0.979	
Final R indices [I > 2sigma(I)]	R_1 = 0.1256, wR_2 = 0.2748	
R indices (all data)	R_1 = 0.1263, wR_2 = 0.2752	
Extinction coefficient	0.0008(3)	
Largest diff. peak and hole	1.108 and -0.393 e $Å^{-3}$	

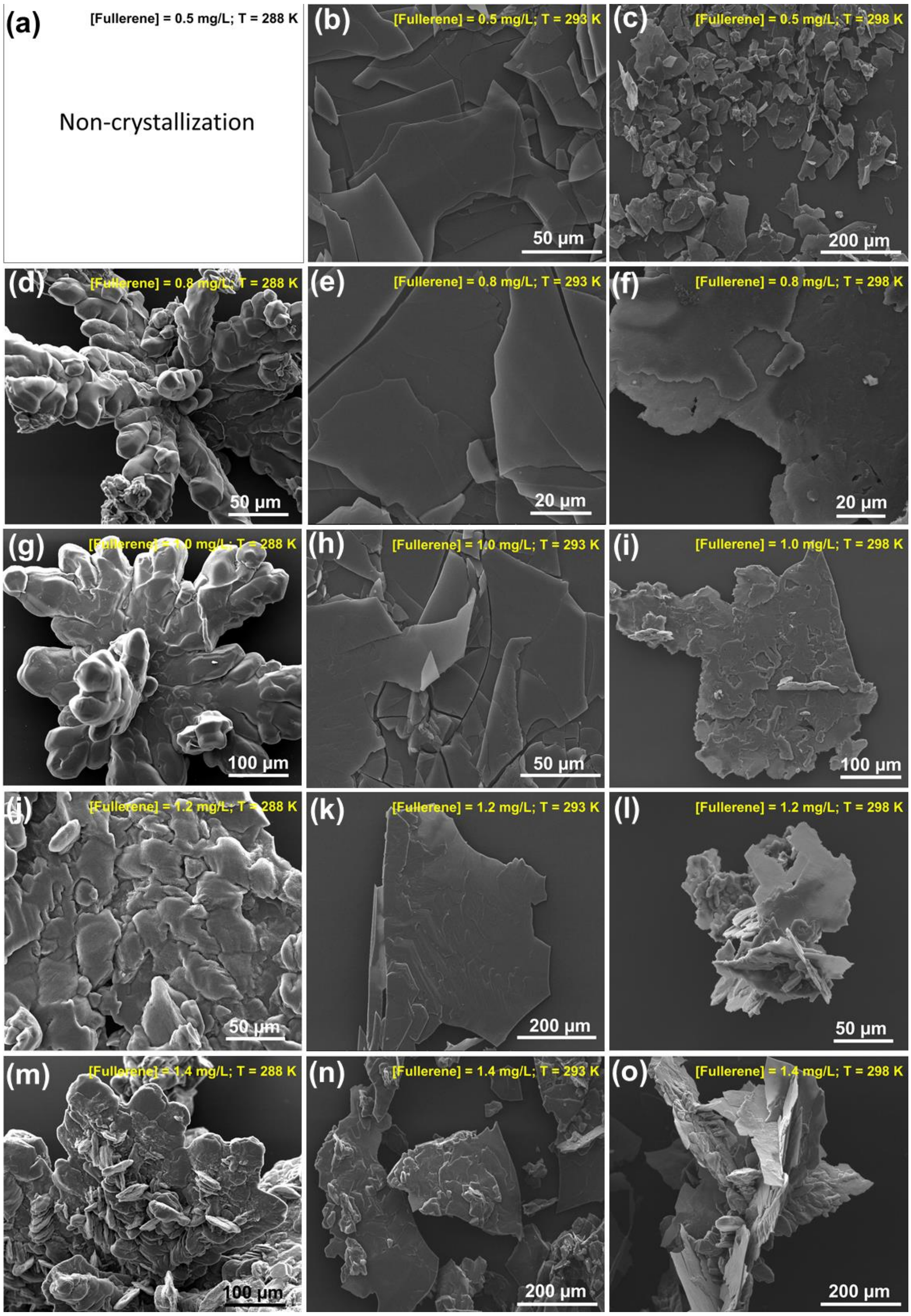

Figure S5. Effects of the fullerene concentration and temperature on fullerene crystallisation ([gelator] = 1.0 % (w/v), [fullerene] = 0.5, 0.8, 1.0, 1.2, 1.4 mg/mL, T= 288, 273, 298 K; Note: (a) non-crystallisation, [fullerene] = 0.5 mg/mL, T= 288 K).

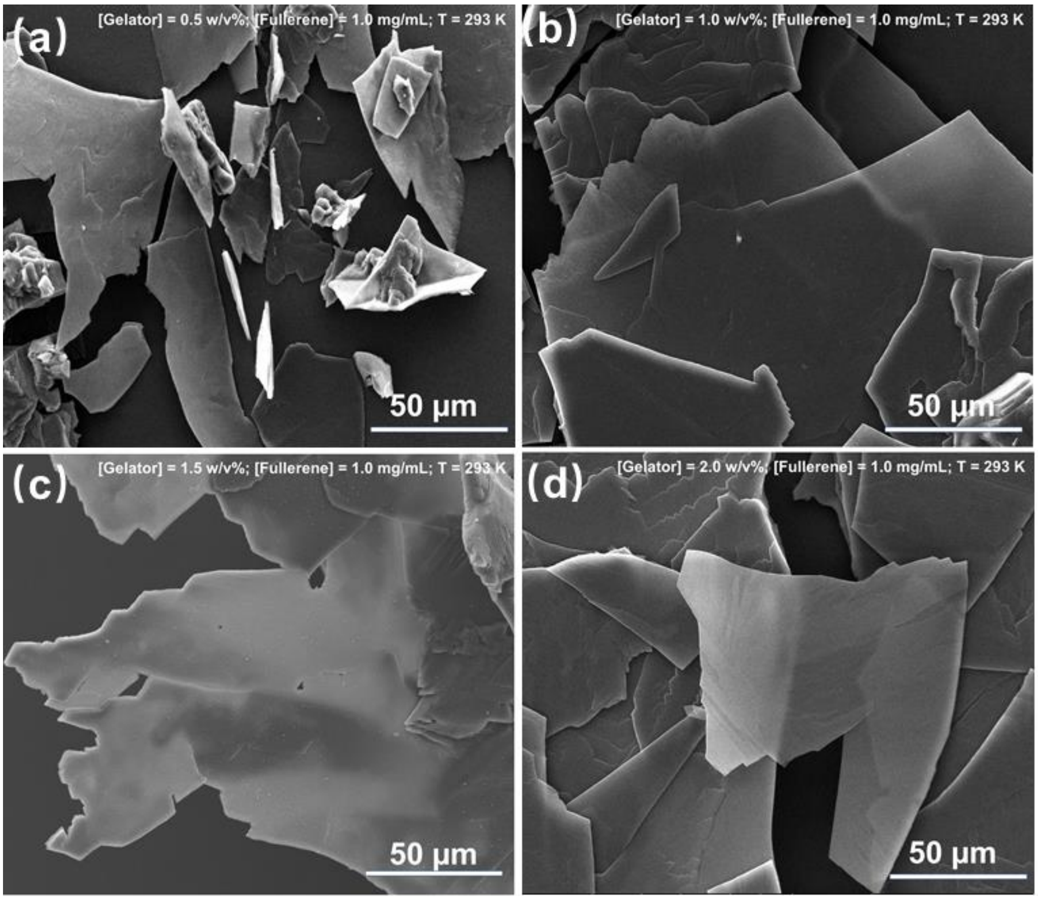

Figure S6. Effect of the gelator concentration on fullerene crystallisation ([fullerene] = 1.0 mg/mL, [gelator] = 0.5, 1.0, 1.5, 2.0% w/v)

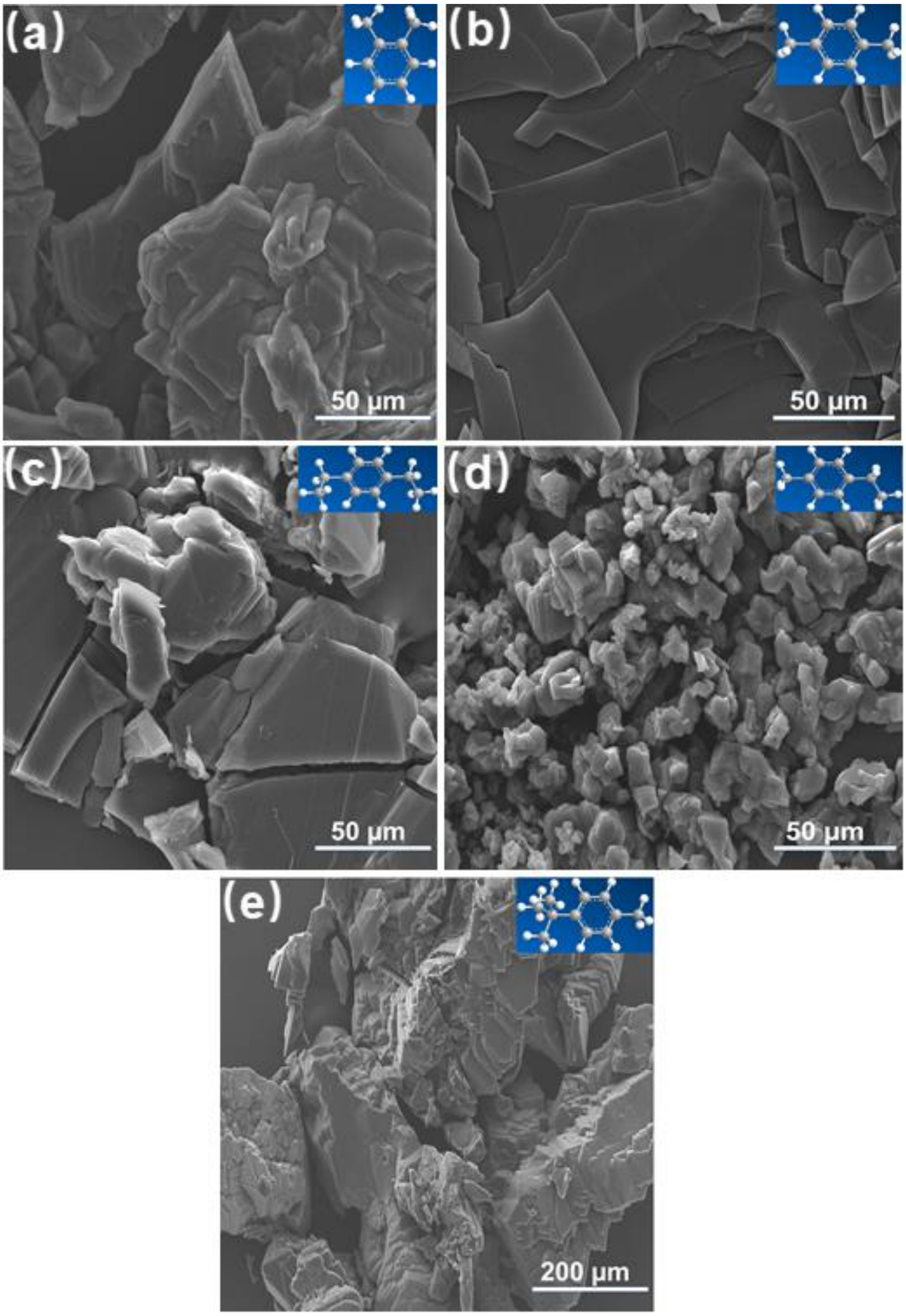

Figure S7. SEM images of the separated crystals induced by acetonitrile in the gels with various gelated solvents ([C_{60}] = 0.5 mg/L; [gelator **1**] = 1.0 % w/v).

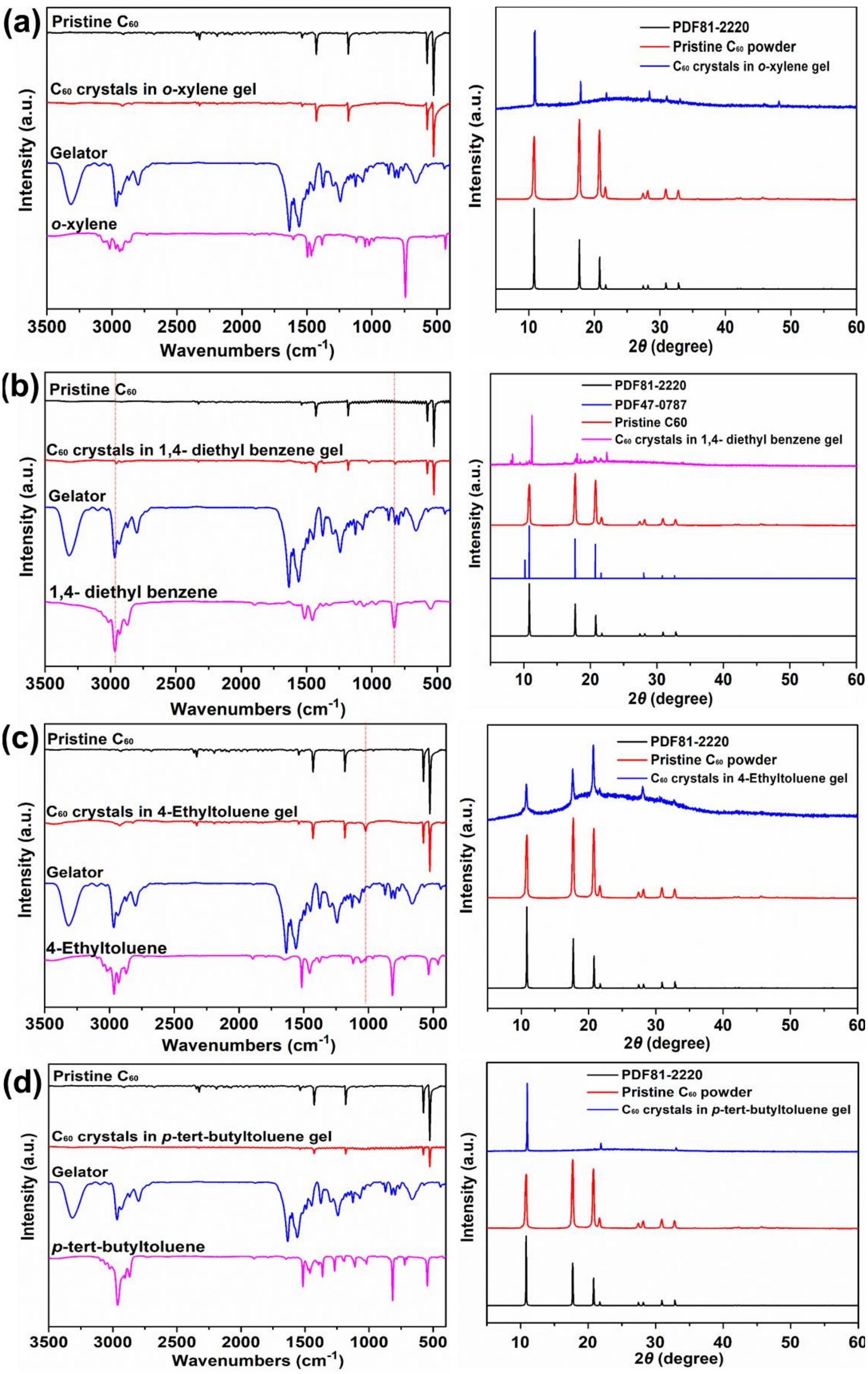

Figure S8. IR spectra and XRD patterns corresponding to the separated crystals in Figure S4 ([C_{60}] = 0.5 mg/L; [gelator 1] = 1.0 % w/v): (a) without solvent inserted, *fcc*; (b) with solvent inserted, almost *hcp*; (c) with solvent inserted, *fcc*; (d) without solvent inserted, *fcc*.

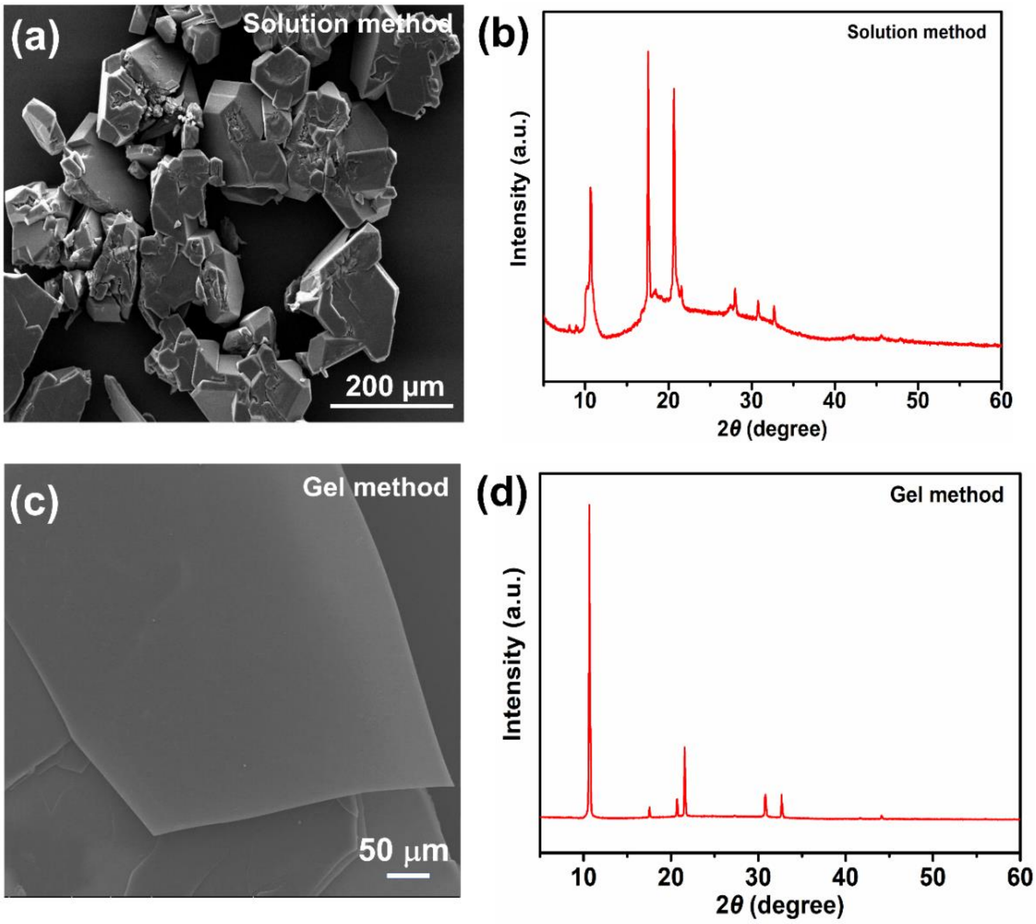

Figure S9. Function of the supramolecular gel in 2D fullerene crystallisation: (a, b) crystal morphology and XRD spectrum of fullerene obtained using the solution method; (c, d) crystal morphology and XRD spectrum of fullerene obtained using the supramolecular gel approach.

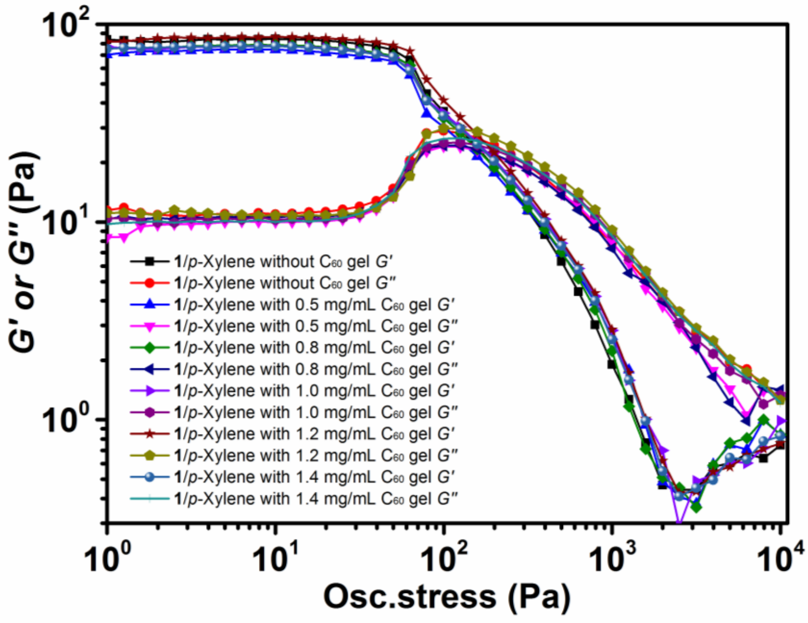

Figure S10. Rheological behaviour of supramolecular gels with various fullerene concentrations (stress sweep at a fixed frequency of 1 Hz at 20 °C, [gelator **1**] = 1% w/v, [fullerene] = 0, 0.5, 0.8, 1.0, 1.2, 1.4 mg/mL).

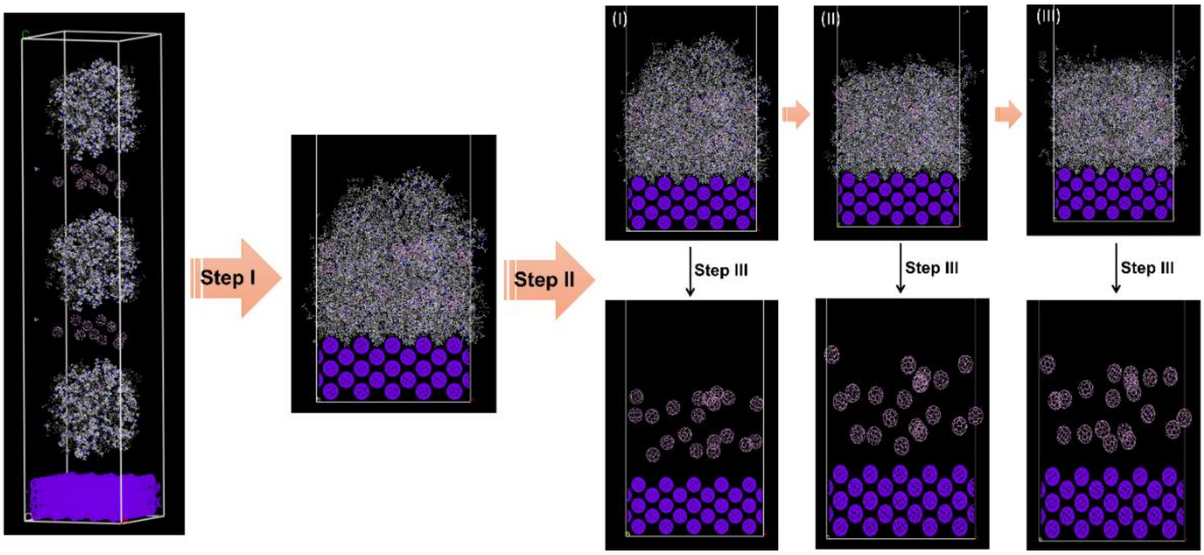

Figure S11. Fullerene crystallisation in supramolecular gel: (Step I) all components are mixed to form a single phase on the crystal plane; (Step II) three-step mixing (force-induced); (Step III) removal of gelators and solvents.

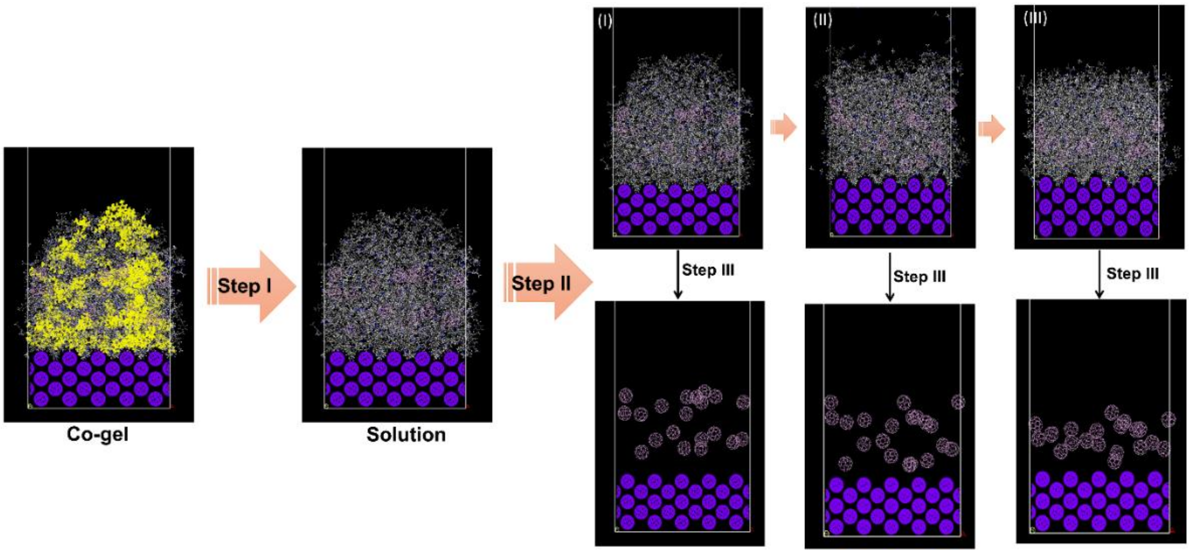

Figure S12. Fullerene crystallisation in supramolecular gel: (Step I) removal of gelators from the co-gel phase above the crystal plane; (Step II) three-step mixing (force-induced); (Step III) removal of solvents.

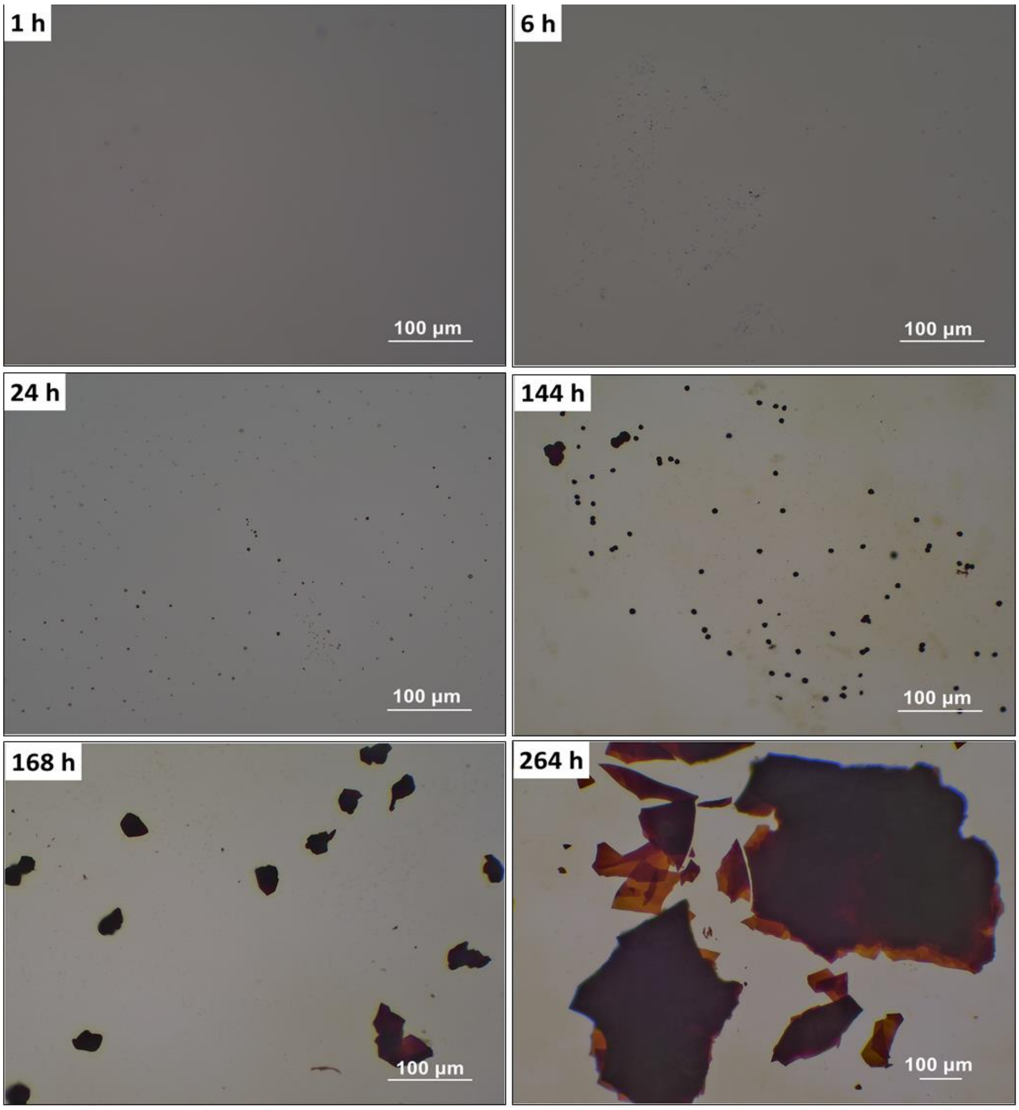

Figure S13. Crystallisation of fullerene molecules from nuclei into 2D crystals (Time: 1 h to 26 h, [fullerene] = 0.5 mg/mL, [gelator] = 1% w/v, temperature = 20 °C).

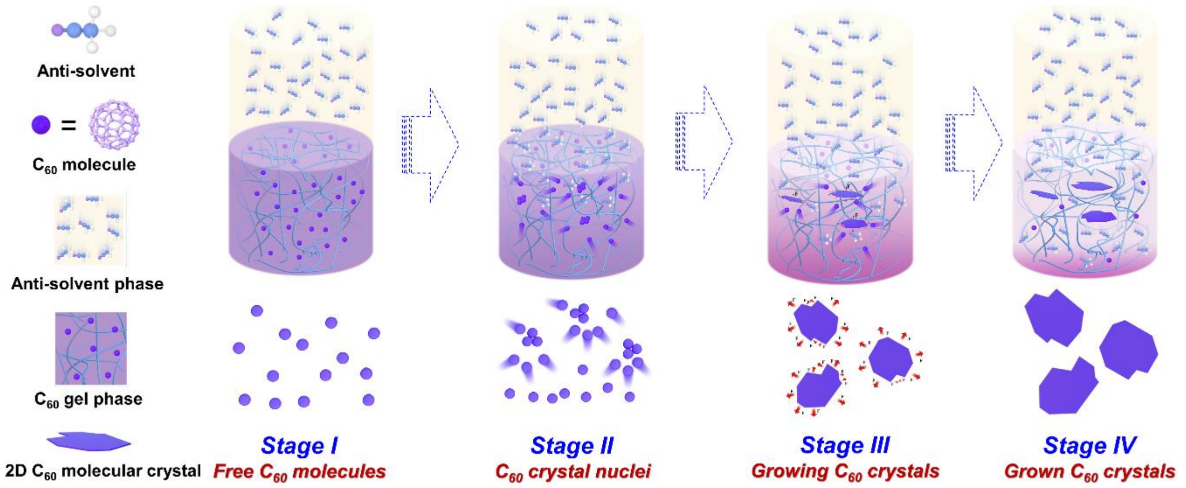

Figure S14. Schematic of the proposed 2D crystallisation process in supramolecular gel triggered by the evaporation of an anti-solvent.

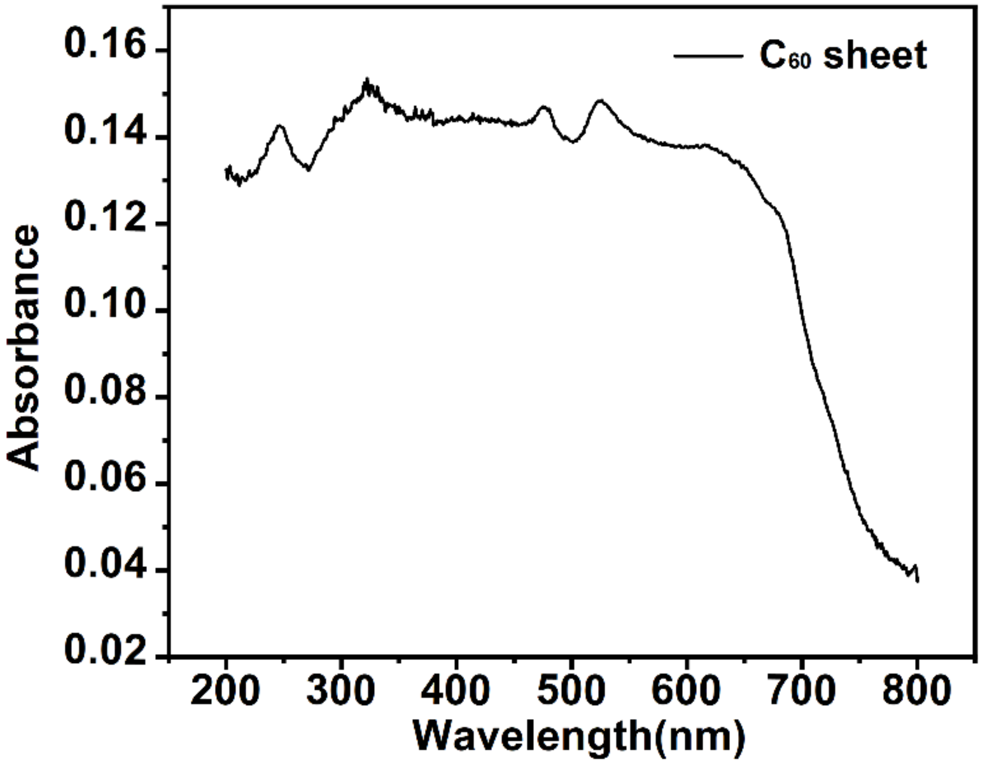

Figure S15. Solid UV spectrum of 2D C_{60} crystals.

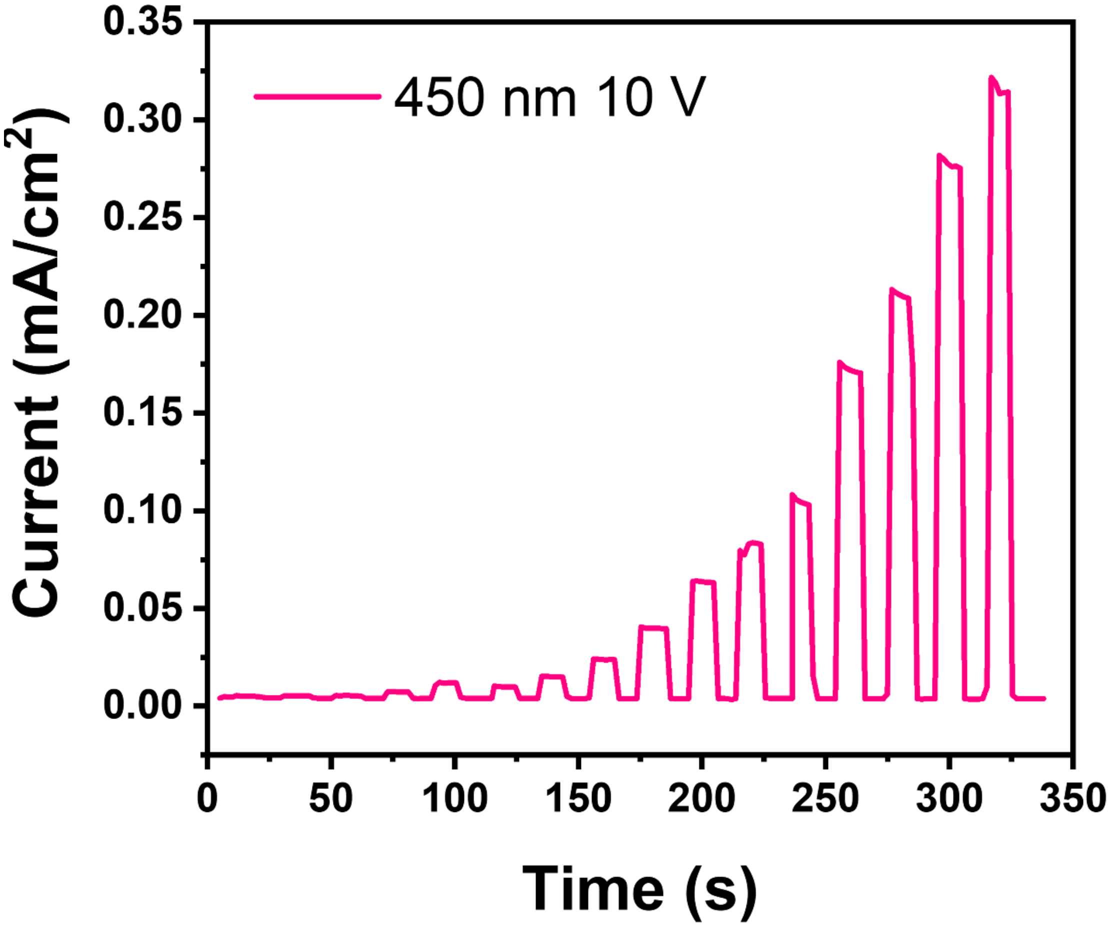

Figure S16. The time-dependent on and off photoresponse measurements of the 2D C_{60} device under 450 nm when the light intensity increases from 4.50×10^{-3} mW/cm^2 to 7.76 mW/cm^2.

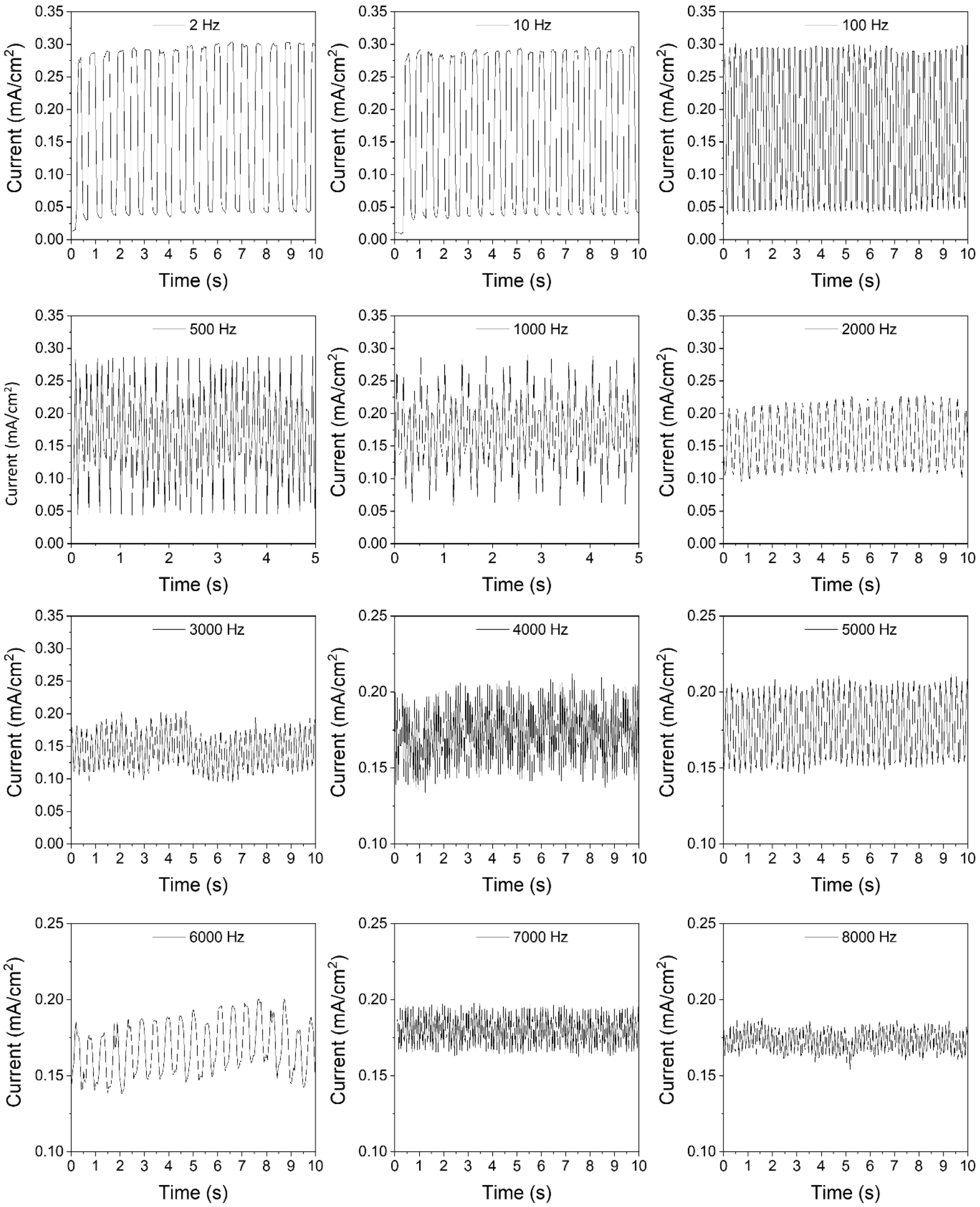

Figure S17. The photoresponse of 2D C_{60} device measured at frequency ranging from 2 Hz to 8000 Hz.

Table S3. Comparison of 2D fullerene crystallisation using various growth methods

No.	Crystallisation method	Height	Lateral size	Application
This study	Growth in supramolecular gels	3–5 nm	1.0×10^5 μm^2	(Opto)electronics devices
1	Liquid−liquid interfacial precipitation method (LLIP)	0.2–2 μm	4500 μm^2	Electrochemical Supercapacitors
2	Supramolecular template method	Not mentioned	Not mentioned	Not mentioned
3	Liquid–liquid interface	0.2 μm	0.16–27 μm^2	Not mentioned
4	Solvent evaporation method	5–6 nm	10 μm^2	2D supramolecular liquid crystals
5	Cooling crystallisation method	6 nm	15 μm^2	Photovoltaic and OFET devices
6	Solvent and template method	Not mentioned	Not mentioned	Photovoltaic and OFET devices
7	Chemical deposition method	2 nm	25–160 nm^2	Superconductors
8	Template method	5 nm	20.0 μm^2	Photovoltaic and OFET devices
Note	The corresponding references are given below.			

References for Table S3:

[1]. Q. Tang, P. Bairi, R. G. Shrestha, J. P. Hill, K. Ariga, H. Zeng, Q. M. Ji, L. K. Shrestha, *Appl. Mater. Interfaces* **2017**, *9*, 44458.

[2]. M. Li, K. Deng, S. B. Lei, Y. L. Y ang, T. S. Wang, Y. T. Shen, C. R. Wang, Q. D. Zeng, C. Wang, *Angew. Chem. Int. Ed.* **2008**, *47*, 6717.

[3]. M. Sathish, K. Miyazawa, *J. Am. Chem. Soc.* **2007**, *129*, 13816.

[4]. X. Zhang, C. H. Hsu, X. Ren, Y. Gu, B. Song, H. J. Sun, S. Y ang, E. Chen, Y. Tu, X. Li, *Angew. Chem. Int.* Ed. **2015**, *54*, 114.

[5]. Y. Hu, K. Yi. Wu, T.T. Zhu, P. Shen, Y. Zhou, X. H. Li, C. L. Wang, Y. F. Tu, C. Y. Li, *Angew. Chem. Int. Ed.* **2018**, *57*, 13454.

[6]. D. L. Cui, M. Ebrahimi, F. Rosei, J. M. Macleod, *J. Am. Chem. Soc.* **2017**, *139*, 16732.

[7]. V. Zotov, D. A. Olyanich, V. V. Mararov, T. V. Utas, L. V. Bondarenko, A. Y. Tupchaya, D. V. Gruznev, A. N. Mihalyuk, C. M. Wei, Y. L. Wang, A. A. Saranin, *J. Chem. Phys.* **2018**, *149*, 034702.

[8]. K. Lee, B. Choi, I. J. Pante, M. V. Paley, X. J. Zhong, A. C. Crowther, J. S. Owen, X. Y. Zhu, X. Roy, *Angew. Chem. Int. Ed.* **2018**, *57*, 6125.

Table S4. Comparison of photoelectric properties for the 2DOMC-based devices under visible light illumination

2D Molecular Crystals	Synthesizing strategy	Responsivity (light wavelength, intensity)	Detectivity	On-off ratio	Reference
ZnTPP-C_{60} cocrystals (thin film)	Solution-processed method	2.23 A/W (450 nm)	7.94 x 10^{11} Jones	NA	1
Supramolecular Fullerene liquid crystals	Cooling solution crystallization method	NA	NA	NA	2, 3, 4
Graphene/C_{60}	Graphene template-induced crystallization method	~10^7 A/W (UV)	NA	NA	5
PbPc : C_{60} Blend Film	Thermal evaporation deposition	244 mA/W	1.36 × 10^{11} Jones	NA	6
NiTPP- C_{60}	Liquid-bridge induced assembly method	11.9 A/W at 425 nm	NA	NA	7
C_{60}-pentacene	LLIP method	NA	NA	NA	8
C_{60} nanosheet (3-5 nm)	Spatially Confined Growth in a Supramolecular Gel	41.2 mA/W (450 nm, 7.76 mW/cm^2)	2.9 x 10^{11} Jones	147	This work
Note	The corresponding references are given below.				

References for Table S4:

1. Y. Wang, H. Wu, W. G. Zhu, X. T. Zhang, Z. Y. Liu, Y. S. Wu, C. F. Feng, Y. F. Dang, H. L. Dong, H. B. Fu, W. P. Hu, *Angew. Chem. Int. Ed.* **2021**, *60*, 6344.
2. X. L. Liu, X. G. Luo, H. Y. Nan, H. Guo, P. Wang, L. L. Zhang, M. M. Zhou, Z. Y. Yang, Y. Shi, W. D. Hu, Z. H. Ni, T. Qiu, Z. F. Yu, J. B. Xu, X. R. Wang, *Adv. Mater.* **2016**, *28*, 5200.
3. X. Y. Zhang, C. H. Hsu, X. K. Ren, Y. Gu, B. Song, H. J. Sun, S. Yang, E. Q. Chen, Y. F. Tu, X. H. Li, X. M. Yang, Y. W. Li, X. L. Zhu, *Angew. Chem. Int. Ed.* **2015**, *54*, 114.
4. Y. Hu, K. Yi. Wu, T. T. Zhu, P. Shen, Y. Zhou, X. H. Li, C. L. Wang, Y. F. Tu, C. Y. Li, *Angew. Chem. Int. Ed.* **2018**, *57*, 13454.
5. S. C. Qin, X. Q. Chen, Q. Q. Du, Z. H. Nie, X. R. Wang, H. Lu, X. Z. Wang, K. H. Liu, Y. B. Xu, Y. Shi, R. Zhang, F. Q. Wang, *ACS Appl. Mater. Interfaces* **2018**, *10*, 38326.
6. M. S. Choi, S. M. Chae, H. J. Kim, J. J. Kim, *ACS Appl. Mater. Interfaces* **2018**, *10*, 25614.
7. M. M. Su, Y. J. Hu, A. Yu, Z. Y. Peng, W. T. Long, S. X. Gao, P. Peng, B. Su, F. F. Li, *Nanoscale Adv.* **2021**, *3*, 1096.
8. Q. Tang, G. P. Zhang, B. H. Jiang, D. Y. Ji, H. H. Kong, K. Riehemann, Q. M. Ji, H. Fuchs, *SmartMat.* **2021**, *2*, 109.
